# A SARS-CoV-2-specific CAR-T-cell model identifies felodipine, fasudil, imatinib, and caspofungin as potential treatments for lethal COVID-19

**DOI:** 10.1038/s41423-023-00985-3

**Published:** 2023-03-02

**Authors:** Lin Xia, Lun-zhi Yuan, Ya-hong Hu, Jun-yi Liu, Guo-sheng Hu, Ruo-yao Qi, Tian-ying Zhang, Hua-long Xiong, Zao-zao Zheng, Hong-wei Lin, Jia-mo Zhang, Chao Yu, Ming Zhou, Jian Ma, Tong Cheng, Ri-rong Chen, Yi Guan, Ning-shao Xia, Wen Liu

**Affiliations:** 1 State Key Laboratory of Cellular Stress Biology, School of Pharmaceutical Sciences, Xiamen University, Xiang’an South Road, Xiamen, Fujian 361102 China; 2 Fujian Provincial Key Laboratory of Innovative Drug Target Research, School of Pharmaceutical Sciences, Xiamen University, Xiang’an South Road, Xiamen, Fujian 361102 China; 3 State Key Laboratory of Molecular Vaccinology and Molecular Diagnostics, National Institute of Diagnostics and Vaccine Development in Infectious Diseases, School of Life Sciences, School of Public Health, Xiamen University, Xiang’an South Road, Xiamen, Fujian 361102 China; 4 State Key Laboratory of Emerging Infectious Diseases, School of Public Health, Li Ka Shing Faculty of Medicine, The University of Hong Kong, Hong Kong SAR, China

**Keywords:** COVID-19, SARS-CoV-2, CAR-T, anti-inflammation, NF-κB pathway, Chronic inflammation, Viral infection, High-throughput screening

## Abstract

Severe acute respiratory syndrome coronavirus 2 (SARS-CoV-2)-induced cytokine storm is closely associated with coronavirus disease 2019 (COVID-19) severity and lethality. However, drugs that are effective against inflammation to treat lethal COVID-19 are still urgently needed. Here, we constructed a SARS-CoV-2 spike protein-specific CAR, and human T cells infected with this CAR (SARS-CoV-2-S CAR-T) and stimulated with spike protein mimicked the T-cell responses seen in COVID-19 patients, causing cytokine storm and displaying a distinct memory, exhausted, and regulatory T-cell phenotype. THP1 remarkably augmented cytokine release in SARS-CoV-2-S CAR-T cells when they were in coculture. Based on this “two-cell” (CAR-T and THP1 cells) model, we screened an FDA-approved drug library and found that felodipine, fasudil, imatinib, and caspofungin were effective in suppressing the release of cytokines, which was likely due to their ability to suppress the NF-κB pathway in vitro. Felodipine, fasudil, imatinib, and caspofungin were further demonstrated, although to different extents, to attenuate lethal inflammation, ameliorate severe pneumonia, and prevent mortality in a SARS-CoV-2-infected Syrian hamster model, which were also linked to their suppressive role in inflammation. In summary, we established a SARS-CoV-2-specific CAR-T-cell model that can be utilized as a tool for anti-inflammatory drug screening in a fast and high-throughput manner. The drugs identified herein have great potential for early treatment to prevent COVID-19 patients from cytokine storm-induced lethality in the clinic because they are safe, inexpensive, and easily accessible for immediate use in most countries.

## Introduction

The rapid emergence and dissemination of severe acute respiratory syndrome coronavirus 2 (SARS-CoV-2) and the subsequent coronavirus disease 2019 (COVID-19) pandemic has placed an excessive burden on public and private health care systems, with over 6,200,000 deaths worldwide (https://www.who.int/). Accumulating evidence suggests that the severe complications and poor outcomes of COVID-19 are strongly associated with cytokine storm (CS) [1–3]. Cytokine storm is a rapidly developing and life-threatening clinical condition in which the overproduction of inflammatory cytokines and excessive activation of immune cells lead to complicated medical syndromes from persistent fever, nonspecific muscle pain, and hypotension to capillary leak syndrome, disseminated intravascular coagulation (DIC), acute respiratory distress syndrome (ARDS), hemophagocytic lymphohistiocytosis (HLH), multiorgan failure, and death if treatment is not adequate [2, 4, 5]. Recent studies have also shown that COVID-19 patients, including children and adults, frequently exhibit multisystem inflammatory syndrome (MIS) [6, 7]. Although rare, MIS causes severe complications in which different body parts become inflamed, including the heart, brain, lungs, liver, skin, eyes, and kidneys [6–8]. Current anti-inflammatory treatments, such as corticosteroids, chloroquine, and colchicines [5, 9], often exhibit side effects postrecovery in patients with nonsevere COVID-19. Tocilizumab, a monoclonal antibody (mAb) targeting IL6R used for rheumatic conditions and CAR-T-cell therapy-induced cytokine storm, is being utilized for treating COVID-19 patients [5, 10, 11]. However, the inhibition of a single cytokine is not sufficient, as multiple cytokines are involved in triggering cytokine storm [12, 13]. Cytokine storm has also been diagnosed in many other inflammatory diseases, such as autoimmune conditions and organ transplantation [5, 14–16]. We hypothesized that a CAR-T-cell-based system might be used to mimic SARS-CoV-2-specific immune responses, providing a tool for screening drugs that can suppress SARS-CoV-2-induced inflammation to treat COVID-19.

For this purpose, we generated a SARS-CoV-2 spike (S) protein-targeted CAR construct and infected T lymphocytes to establish a SARS-CoV-2-specific CAR-T-cell model. Upon incubation with 293 T cells expressing the full-length S protein (S-293T), SARS-CoV-2-specific CAR-T cells were activated, releasing a large amount of cytokines and displaying profiles similar to those of the T cells observed in COVID-19 patients. Based on this model, an FDA-approved drug library was screened, which led to the discovery that one calcium antagonist, felodipine; one Rho kinase inhibitor, fasudil; one tyrosine kinase inhibitor, imatinib; and one antifungal inhibitor, caspofungin, were effective in suppressing cytokine release. Using a SARS-CoV-2-infected, lethal Syrian hamster model, we leveraged the ability to perform longitudinal collections to test the pathologic, immunologic, and virologic effects of these drugs, revealing that they were effective in attenuating lethal inflammation, ameliorating severe pneumonia, and rescuing mortality.

## Results

### Construction of SARS-CoV-2 S protein-targeted CAR-T cells

**Fig. 1:**
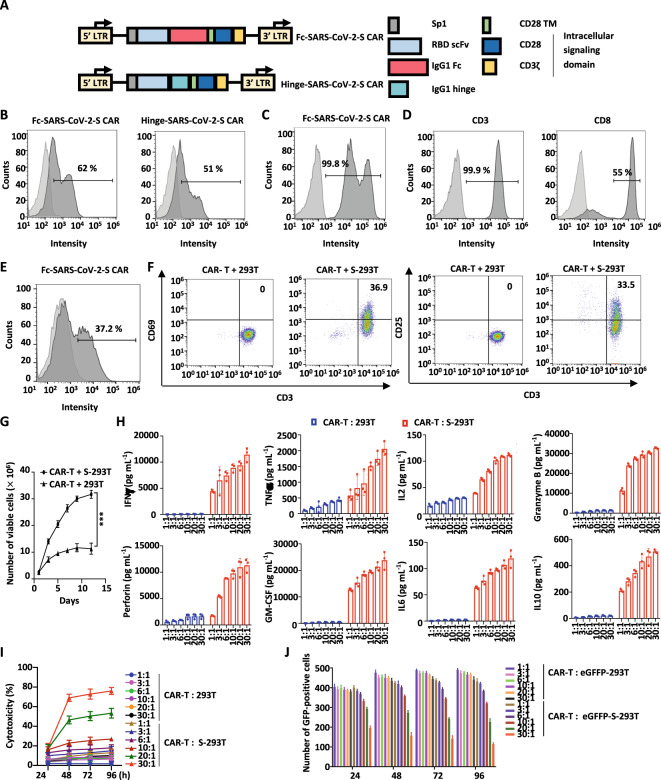
The expression, activation, and function of SARS-CoV-2 S protein-targeted CAR-T cells (SARS-CoV-2-S CAR-T). **A** Schematic illustration of two second-generation SARS-CoV-2-S CAR constructs. The CAR is composed of a signal peptide of the interleukin (IL)2 receptor (Sp1), an anti-RBD scFv, a spacer (IgG1 Fc or IgG1 hinge), a CD28 transmembrane domain (CD28 TM), and intracellular signaling domains (CD28 and CD3ζ). The spacers IgG1 Fc and IgG1 hinge were used in Fc-SARS-CoV-2-S and Hinge-SARS-CoV-2-S CAR constructs, respectively. **B** 293 T cells transfected with a control lentiviral vector (CTL vector) or SARS-CoV-2-S CAR expression vectors as described in **A** were stained with eGFP-tagged S protein followed by flow cytometry analysis. Light gray, CTL vector; dark gray, SARS-CoV-2-S CAR. **C** 293 T cells transfected with a CTL vector or Fc-SARS-CoV-2-S CAR expression vector as described in **A** were stained with an APC-conjugated anti-human IgG-Fc antibody followed by flow cytometry analysis. Light gray, CTL vector; dark gray, Fc-SARS-CoV-2-S CAR. **D** Primary T lymphocytes from a healthy donor were expanded and stained with an anti-CD3 antibody conjugated with FITC (CD3-FITC) (left panel) or an anti-CD8 antibody conjugated with APC (CD8-APC) (right panel) followed by flow cytometry analysis. Light gray, blank; dark gray, CD3 or CD8 staining. **E** Primary T lymphocytes infected with control lentivirus (CTL lentivirus) or Fc-SARS-CoV-2-S CAR lentivirus as described in **A** were stained with an APC-conjugated anti-human IgG-Fc antibody followed by flow cytometry analysis. Light gray, CTL lentivirus; dark gray, Fc-SARS-CoV-2 CAR-S lentivirus. **F** SARS-CoV-2-S CAR-T cells were incubated with 293 T cells or 293 T cells transfected with S protein (S-293T cells) at a ratio of 3:1 for two days, and T cells in suspension were separated from adherent 293 T cells and costained with CD3-APC and CD69-PE or CD25-FITC followed by flow cytometry analysis. **G** SARS-CoV-2-S CAR-T cells were incubated with 293 T or S-293T cells and maintained in culture medium for the indicated durations. The number of viable cells was counted at the indicated time points (mean ± s.e.m, ***P < 0.001). **H** SARS-CoV-2-S CAR-T cells were incubated with 293 T or S-293T cells at different ratios (1:1, 3:1, 6:1, 10:1, 20:1 or 30:1) for three days before measuring the secretion of cytokines, including IFNγ, TNFα, IL2, granzyme B, perforin, GM-CSF, IL6, and IL10. CTL T cells were used as a negative control (mean ± s.e.m). **I** SARS-CoV-2-S CAR-T cells were incubated with 293 T or S-293T cells at different ratios for the indicated durations, followed by a cytotoxicity assay (mean ± s.e.m). **J** SARS-CoV-2-S CAR-T cells were incubated with 293 T cells fused with eGFP (eGFP-293T) or 293 T cells expressing spike fused with eGFP (eGFP-S-293T) at different ratios for the indicated duration, followed by GFP fluorescence detection. The number of GFP-positive cells was counted (mean ± s.e.m)

To construct the S protein-targeted CAR, the antigen recognition part of CAR was engineered using variable heavy and light chain sequences from an anti-RBD antibody targeting the S protein, which was reported by us previously [17] (Fig. 1A). This anti-RBD antibody also showed significant neutralizing activity toward the mutated S protein from the SARS-CoV-2 Delta variant (IC_50_ = 21.12 ng/mL) (Fig. S1A). Two forms of IgG1-based spacers, the IgG1 Fc spacer and the IgG1 hinge spacer, were tested in CAR construction (Fc-SARS-CoV-2-S CAR and Hinge-SARS-CoV-2-S CAR) (Fig. 1A). Flow cytometry analysis results showed that Fc-SARS-CoV-2-S CAR recognized the S protein more efficiently than Hinge-SARS-CoV-2-S CAR when expressed in 293 T cells (Fig. 1B). The Fc-SARS-CoV-2 CAR construct was highly expressed (99.8%) in 293 T cells, as identified by an anti-human IgG-Fc antibody (Fig. 1C). Primary human T cells from a healthy donor were enriched and expanded in vitro in the presence of anti-CD3/CD28 monoclonal antibodies, IL2, and IL15 for one week. Approximately 55% of these T cells were CD3^+^ CD8^+^ as examined by flow cytometry (Fig. 1D). Enriched T cells were then infected with lentivirus encoding Fc-SARS-CoV-2-S CAR, which were referred to as Fc-SARS-CoV-2-S CAR-T (SARS-CoV-2-S CAR-T for short) cells. The infection efficiency of SARS-CoV-2-S CAR-T cells was 37.2% (Fig. 1E). Both CD69^+^/CD3^+^ and CD25^+^/CD3^+^ subpopulations increased more than 33% in SARS-CoV-2-S CAR-T cells upon coincubation with 293 T cells expressing S protein (S-293T cells) compared to control 293 T cells (Fig. 1F). When expanded under the T-cell culture protocol, the number of SARS-CoV-2-S CAR-T cells increased much faster when cocultured with S-293T cells than parental 293 T cells in two weeks (Fig. 1G). To further demonstrate the specific activation of SARS-CoV-2-S CAR-T cells by the S protein, we introduced the mutated S protein from the SARS-CoV-2 variant Omicron into 293 T cells (Omicron-293T). This mutated S protein could not be recognized by the anti-RBD antibody used in our CAR-T model (Fig. S1A). Our results indicated that there were no significant differences in T-cell activation or proliferation between the CAR-T cells incubated with control 293 T and Omicron-293T cells, suggesting that antigen-specific stimulation played a critical role in activating SARS-CoV-2-S CAR-T cells (Fig. S1B, C). Collectively, SARS-CoV-2-S CAR-T cells can be specifically activated by the SARS-CoV-2 S protein.

To evaluate the cytokines produced by the activated CAR-T cells, we cocultured SARS-CoV-2-S CAR-T cells with S-293T cells and found that SARS-CoV-2-S CAR-T cells secreted much higher levels of cytokines, such as IFNγ, TNFα, IL2, perforin, granzyme B, GM-CSF, IL6 and IL10, when cocultured with S-293T cells than when cocultured with control 293 T cells (Fig. 1H). Notably, the production of IFNγ, perforin, granzyme B, and GM-CSF reached a considerably high level (10,000–20,000 pg/mL) at the highest dose of SARS-CoV-2-S CAR-T cells. To confirm that the cytokines released were from CAR-T cells, we collected SARS-CoV-2-S CAR-T and S-293T cells separately after coincubation to measure the expression of representative cytokines by RT‒qPCR analysis. Our results showed that the levels of IFNγ and IL6 increased significantly in activated CAR-T cells (Fig. S2A). In contrast, the induction of IFNγ and IL6 was very limited in S-293T cells (Fig. S2B).

Next, we sought to examine the cytotoxicity of SARS-CoV-2-S CAR-T cells by incubating SARS-CoV-2-S CAR-T cells with S-293T cells or control 293 T cells at different ratios and durations. The cytotoxicity of SARS-CoV-2-S CAR-T cells did not show a dramatic change even when tenfold more CAR-T cells than S-293T cells were added, and strong cytotoxicity was observed only at extremely high doses of CAR-T cells (20:1 or higher) (Fig. 1I). In addition, as we extended the duration of treatment, the cytotoxicity of SARS-CoV-2-S CAR-T cells did not show a dramatic change, which was different from what we observed for other CAR-T cells [18]. These results suggested that SARS-CoV-2-S CAR-T cells were inert in response to antigen exposure (Fig. 1I), which was independently confirmed by counting the number of GFP-positive cells in 293 T cells expressing an eGFP reporter (eGFP-S-293T) (Fig. 1J and Fig. S3).

### SARS-CoV-2-S CAR-T cells mimic the gene signatures of T cells in COVID-19 patients

**Fig. 2:**
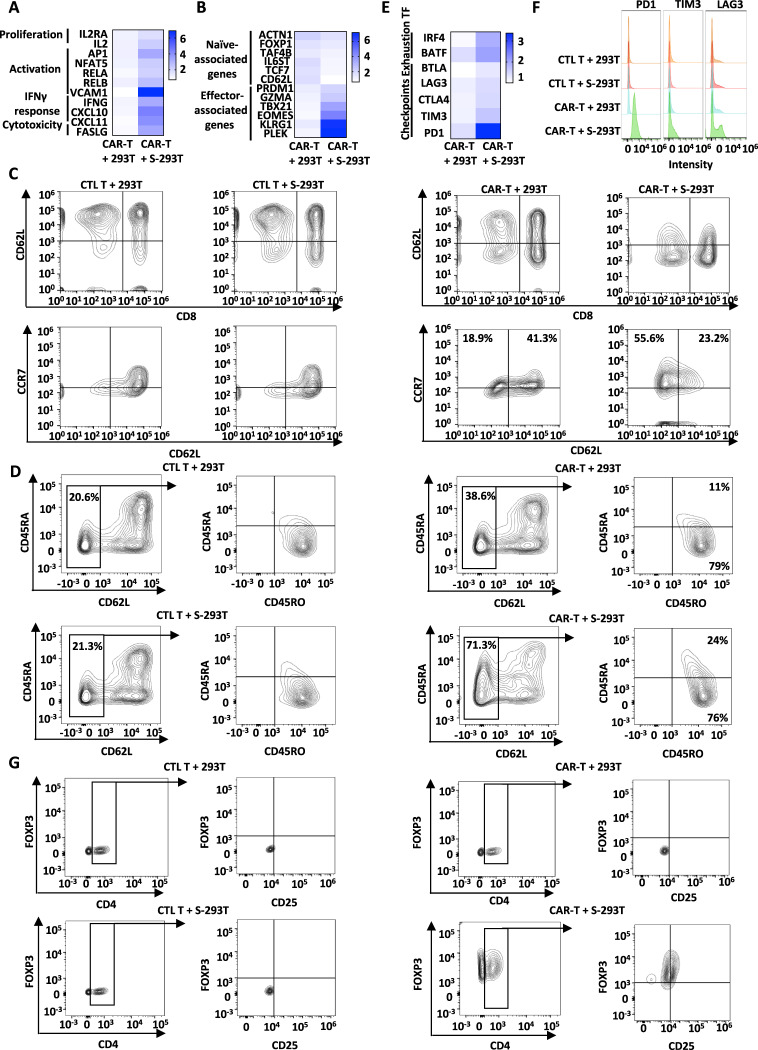
Phenotypical characterization of SARS-CoV-2-S CAR-T cells upon antigen stimulation. SARS-CoV-2-S CAR-T cells were incubated with 293 T or S-293T cells at a ratio of 3:1 for two days, and T cells in suspension were separated from adherent 293 T cells and collected, followed by RNA extraction and RT‒qPCR analysis to examine the expression of representative genes associated with T-cell proliferation, activation, IFNγ response and cytotoxicity (**A**), naive and effector T-cell gene signatures (**B**), and check points and transcription factors (**E**). The significance test is shown in Fig. S2 (*P < 0.05; **P < 0.01; ***P < 0.001). **C** CTL T or SARS-CoV-2-S CAR-T cells were incubated with or without 293 T or S-293T cells at a ratio of 3:1 for two days, and T cells in suspension were separated from adherent 293 T cells and stained with an anti-CD8 antibody conjugated with APC (CD8-APC), an anti-CD62L antibody conjugated with PE (CD62L-PE), and an anti-CCR7 antibody conjugated with FITC (CCR7-FITC) followed by flow cytometry analysis. **D** CAR-T cells as described in **A** were subjected to staining with an anti-CD45RA antibody conjugated with APC (CD45RA-APC), an anti-CD45RO antibody conjugated with FITC (CD45RO-FITC), and an anti-CD62L antibody conjugated with PE (CD62L-PE). Coexpression of CD62L and CD45RA was analyzed by flow cytometry. The expression of CD45RO and CD45RA was further measured on gated CD62L-negative cells. **F** CAR-T cells as described in **A** were subjected to staining with an anti-PD1 antibody conjugated with FITC (PD1-FITC), an anti-TIM3 antibody conjugated with APC (TIM3-APC) and an anti-LAG3 antibody conjugated with PE (LAG3-PE) followed by flow cytometry analysis. **G** CAR-T cells as described in **A** were subjected to staining with an anti-CD25 antibody conjugated with APC (CD25-APC), an anti-CD4 antibody conjugated with PE (CD4-PE) and an anti-FOXP3 antibody conjugated with Alexa Fluor® 488 (FOXP3-488). Coexpression of CD4 and FOXP3 was analyzed by flow cytometry. The expression of CD25 and FOXP3 was further measured on gated CD4-positive cells. All data are representative of three independent experiments

In COVID-19 patients, the T-cell population undergoes quantitative and qualitative changes. The activation of interferon-stimulated genes (ISGs) is frequently observed, particularly in severe disease [19–21]. Our RNA-seq analysis results showed that genes associated with proliferation, T-cell activation, IFNγ response, and cytotoxicity were significantly upregulated in SARS-CoV-2-S CAR-T cells when coincubated with S-293T cells, which was further confirmed by RT‒qPCR analysis (Figs. 2A and S4A). The expression of genes that are naive-associated was markedly decreased in SARS-CoV-2-S CAR-T cells, while genes that are effector-associated were upregulated in activated SARS-CoV-2-S CAR-T cells (Figs. 2B and S4B), which was consistent with a previous report that the percentage of naive T cells was lower in COVID-19 patients [20, 22]. We further examined the expression of the surface markers CD45RA, CD45RO, CD62L, and CCR7 by flow cytometry for CAR-T cells with or without S-293T incubation. A cell population expressing CCR7 but not CD62L (CD62L^-^CCR7^+^) was specifically observed in virus-related memory T cells, which were defined as intermediate memory T cells (T_IM_) [23]. Indeed, a major shift in CAR-T cells from CD62L^+^ to CD62L^-^ and increased expression of CCR7 were observed upon stimulation (Fig. 2C). It is also known that in COVID-19 patients, T_CM_ and T_SCM_ populations are significantly more frequent than in healthy controls [22]. When CD62L-negative cells were gated, the majority of the activated CAR-T-cell population was CD45RO^+^CD45RA^-^ (76%), and the rest was CD45RO^+^CD45RA^+^ (24%) (Fig. 2D, right panels), which are characteristics of central memory T cells (T_CM_, CCR7^+^CD45RO^+^CD45RA^-^) and stem cell memory T cells (T_SCM_, CCR7^+^CD45RO^+^CD45RA^+^), respectively. In contrast, control T cells showed no significant change in the presence of S-293T (Fig. 2C, D, left panels).

In addition, the T-cell compartment displays alterations involving regulatory T cells and exhausted T cells in COVID-19 patients [22, 24]. The exhausted T-cell phenotype and upregulation of inhibitory receptors (IRs), including programmed cell-death 1 (*PD1*) [25], hepatitis A virus cellular receptor 2 (*TIM3*) [26], lymphocyte-activation gene 3 (*LAG3*) [25], cytotoxic T-lymphocyte associated protein 4 (*CTLA4*) [27], and B- and T-lymphocyte-associated protein (*BTLA*) [28], are often observed in patients with COVID-19, particularly in those with severe disease [29, 30]. The expression of these indicators was found to be increased by RT‒qPCR and flow cytometry analysis in SARS-CoV-2-S CAR-T cells coincubated with S-293T cells, with that of PD1 being the most dramatic (Figs. 2E, F and S4C). Furthermore, increased expression of transcription factors (TFs), including interferon regulatory factor 4 *(IRF4)* and basic leucine zipper ATF-like transcription factor (*BATF*), which are known to stimulate the expression of inhibitory receptors and impair effector function [31], was also observed in activated SARS-CoV-2-S CAR-T cells (Figs. 2E and S4C). Within the CD4^+^ population, we analyzed markers commonly related to regulatory T cells (CD4^+^CD25^+^FOXP3^+^) [32]. Compared to controls, we identified 37% of CD4^+^-gated T cells expressing significantly higher levels of FOXP3 after stimulation with S-293T, which were also CD25^+^ (Fig. 2G).

### Monocytes and SARS-CoV-2-S CAR-T cells synergistically induce cytokine storm, with IL8 being the most induced, as seen in COVID-19 patients

**Fig. 3:**
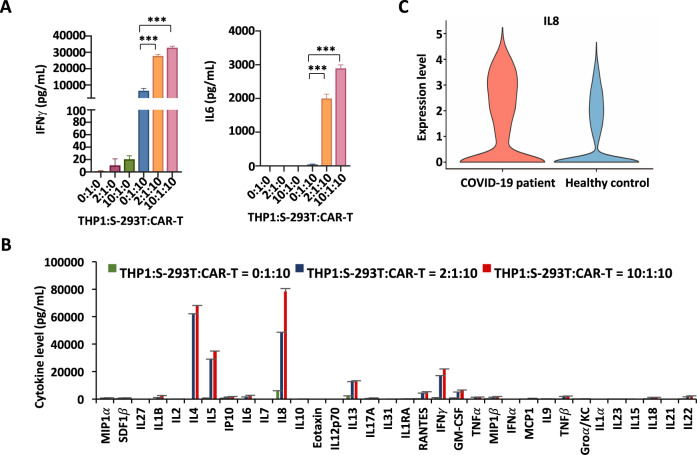
Monocytes enhance the cytokine release of SARS-CoV-2-S CAR-T cells. **A** THP1, S-293T, and SARS-CoV-2-S CAR-T cells were mixed at the indicated ratios for three days before measuring the secretion of IFNγ and IL6 (mean ± s.e.m, ***P < 0.001). Data presented are the mean (± s.e.m) from three repeats. **B** Cells as described in **A** were subjected to measurement of the secretion of multiple cytokines by a multiplex bead array (mean ± s.e.m). **C** The violin plot shows the expression of IL8 (*CXCL8*) in the PBMCs of COVID-19 patients and healthy controls (***P < 0.001)

Monocytes are reported to be critical peripheral sources of cytokine storm in severe COVID-19 patients [33–37]. Monocytes/macrophages and T cells form a positive feedback loop that drives persistent hyperinflammation in SARS-CoV-2 pneumonia [36]. To better mimic SARS-CoV-2-induced cytokine storm, we added human monocyte THP1 cells to SARS-CoV-2-S CAR-T cells coincubated with S-293T cells (Fig. 3A). The best cytokine production, exemplified by IFNγ and IL6, was observed when THP1, CAR-T, and S-293T cells were incubated at a ratio of 10:10:1 (Fig. 3A). The augmentation of cytokine production by THP1 cells was confirmed by a multicytokine assay for a large number of cytokines (Fig. 3B). IL8 (*CXCL8*) was the most secreted cytokine among all cytokines that exceeded 1000 pg/mL, including IL4, IL5, IFNγ, ΙL13, GM-CSF, RANTES (*CCL5*), IL6, IL22, TNFβ, IP-10 (*CXCL10*), IL1B, MIP-1β (*CCL4*), TNFα, IL18, MIP-1α (*CCL3*), and SDF1α (*CXCL12*) (Fig. 3B). Similarly, SARS-CoV-2-S CAR-T cells coincubated with 293 T cells expressing the mutated S protein from the SARS-CoV-2 Delta variant (Delta-293T) and THP1 cells produced significant amounts of IL8 and IFNγ (Fig. S5A).

We then tested whether our “two-cell” (CAR-T and THP1 cells) model could be activated by BEAS-2B, a normal lung cell line derived from human bronchial epithelium. To enhance the infection efficiency of lentiviruses expressing the S protein in BEAS-2B cells, we first established BEAS-2B cells with ACE2 overexpression (hACE2-BEAS-2B). hACE2-BEAS-2B cells were shown to be effectively infected with S-proteins (S-hACE2-BEAS-2B) (Fig. S5B, C). When SARS-CoV-2-S CAR-T cells were incubated with control hACE2-BEAS-2B or S-hACE2-BEAS-2B cells, the release of cytokines such as IL8 and IFNγ was remarkably augmented, which was further enhanced in the presence of THP1 cells (Fig. S5D).

To determine the clinical significance of our SARS-CoV-2-S CAR-T-cell model, we examined whether the cytokines released in the “two-cell” (CAR-T and THP1 cells) model were related to the cytokine storm (CS) that has been observed in COVID-19 patients. Specifically, we analyzed bulk RNA sequencing (bulk RNA-seq) data from the PBMCs of six patients with severe COVID-19 and three healthy controls [38]. Differential expression analysis results showed that the expression of IL8 in the PBMCs differed significantly between the healthy controls and COVID-19 patients (Fig. 3C).

### Felodipine, fasudil, imatinib, and caspofungin, screened in the SARS-CoV-2-S CAR-T-cell model, reduce cytokine release

**Fig. 4:**
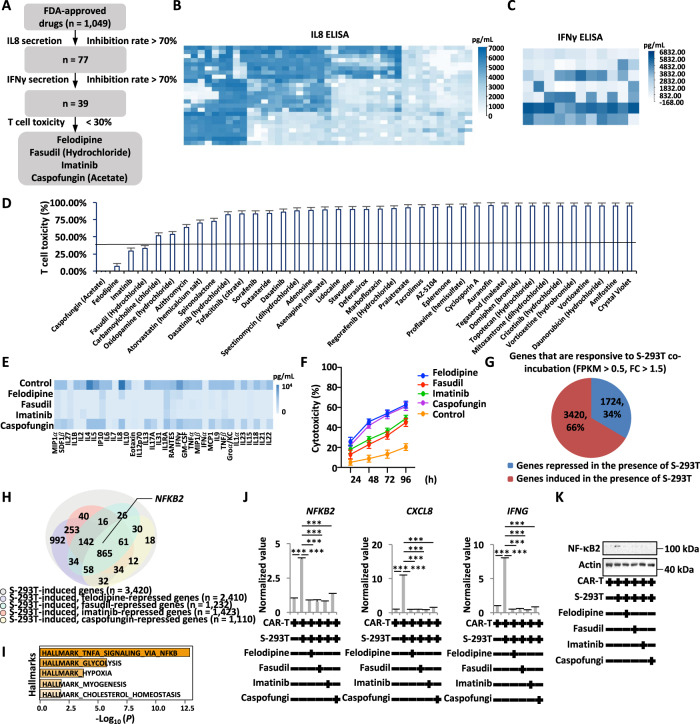
Felodipine, fasudil, imatinib, and caspofungin, screened by the SARS-CoV-2-S CAR-T-cell model, suppress cytokine release. **A** The flowchart for drug screening that can suppress SARS-CoV-2-induced inflammation based on the SARS-CoV-2-S CAR-T-cell model is shown. **B** S-293T, SARS-CoV-2-S CAR-T, and THP1 cells (1:10:10) were coincubated and treated with or without individual drugs (10 μM) from an FDA-approved library (~1049) before measuring the secretion of IL8 in the culture supernatants by ELISA. **C** The culture supernatants from drugs with more than 70% inhibition of IL8 secretion screened from **B** were further subjected to measurement of IFNγ by ELISA. **D** T cells were treated with drugs with more than 70% inhibition of IFNγ secretion screened from **C** for three days followed by toxicity assay (mean ± s.e.m). **E** S-293T, SARS-CoV-2-S CAR-T, and THP1 cells (1:10:10) were coincubated and treated with or without felodipine (#1), fasudil (#2), imatinib (#3), or caspofungin (#4) (10 μM) for three days before measuring the secretion of cytokines by a multiplex bead array. The inhibition rate is presented by a heatmap. **F** SARS-CoV-2-S CAR-T cells coincubated with S-293T cells (5:1) were treated with or without felodipine (#1), fasudil (#2), imatinib (#3), or caspofungin (#4) (10 μM) for the indicated duration, followed by a cytotoxicity assay (mean ± s.e.m). **G** SARS-CoV-2-S CAR-T cells were incubated with control 293 T or S-293T cells at a ratio of 3:1 for two days, and cells in suspension were subjected to RNA-seq analysis. Genes up- and downregulated by S-293T cells are shown in a pie chart (FDR < 0.01, FC > 1.5). **H** SARS-CoV-2-S CAR-T cells incubated with S-293T cells were treated with or without felodipine, fasudil, imatinib, or caspofungin (10 μM) for two days, and cells in suspension were subjected to RNA-seq analysis. Genes that were upregulated by S-293T cells but suppressed by felodipine, fasudil, imatinib, and caspofungin are shown. **I** Hallmark gene set enrichment analysis for overlapping genes (*n* = 865) as described in **H**. **J**, **K** SARS-CoV-2-S CAR-T cells were incubated with S-293T cells and treated with or without felodipine, fasudil, imatinib, or caspofungin (10 μM) for two days. T cells in suspension were subjected to RT‒qPCR analysis (**J**) to examine the expression of genes as indicated or immunoblotting analysis (**K**) using antibodies as indicated (Student’s *t* test, unpaired, two-tailed, *P < 0.05; **P < 0.01; ***P < 0.001)

Having established a cell model system that mimics, at least partially, the cytokine storm in COVID-19 patients, we sought to identify small molecules that can inhibit the release of cytokines. An FDA-approved drug library (*n* = 1049) was tested for its inhibitory effects on the release of the cytokines IL8 and IFNγ by using ELISA (Fig. 4A). The first round of screening for the inhibition of IL8 release led to 77 drugs (Fig. 4A, B), and the second round of screening for the inhibition of IFNγ release resulted in 39 drugs with more than 70% efficiency (Fig. 4A, C). We further tested the T-cell toxicity of these 39 drugs and found that only four of them, caspofungin (acetate), felodipine, imatinib, and fasudil (hydrochloride), exhibited minimal cytotoxicity toward T cells (Fig. 4D). Caspofungin is an antifungal drug [39]. Felodipine is a calcium-channel antagonist [40]. Fasudil is a Rho kinase inhibitor [41–43]. Imatinib is a tyrosine kinase inhibitor [43–45]. The inhibitory effect of these four drugs on cytokine release was further validated using a cytokine panel assay, with felodipine, fasudil, and imatinib exhibiting much more potent activity than caspofungin (Fig. 4E). Consistently, these four drugs could enhance the cytotoxicity of CAR-T cells to S-293T cells (Fig. 4F). These four drugs were also effective in inhibiting cytokine release from SARS-CoV-2-S CAR-T cells incubated with Delta-293T and THP1 cells (Fig. S6A). Similarly, the production of inflammatory cytokines released from SARS-CoV-2-S CAR-T cells incubated with S-hACE2-BEAS-2B and THP1 cells was also inhibited by the four drugs (Fig. S6B).

To understand the anti-inflammatory mechanism of these drugs, SARS-CoV-2-S CAR-T cells and THP1 cells coincubated with S-293T cells were treated with or without drugs, and CAR-T cells were collected and subjected to RNA-seq. The expression of a large cohort of genes in cells was altered when incubated with S-293T cells, with 3420 and 1724 genes being up- and downregulated, respectively (FC > 1.5) (Fig. 4G). In particular, for the 3,420 genes that were induced by S-293T cells, 865 were commonly repressed by these four drugs (Fig. 4H). The most enriched hallmark for these 865 genes was TNFα signaling via NFκB (Fig. 4I). One of the NF-κB subunits, NF-κB2 [46], was among the genes that were induced by S-293T cells and commonly repressed by the four drugs (Fig. 4H). The NF-κB heterodimeric complex (RelB-NF-κB2/p52) is a transcriptional activator [47–50]. We first validated that S-293T-induced NF-κB2 was suppressed by all four drugs through RT‒qPCR analysis (Fig. 4J), and we further confirmed this finding by immunoblotting analysis (Fig. 4K). NF-κB2 downstream target genes [46, 51–53], particularly IL8 (*CXCL8*) and IFNγ (*IFNG*), were also suppressed by drug treatments (Fig. 4J).

### Felodipine, fasudil, imatinib, and caspofungin ameliorate morbidity and severe pneumonia and prevent mortality in SARS-CoV-2-infected hamsters

**Fig. 5:**
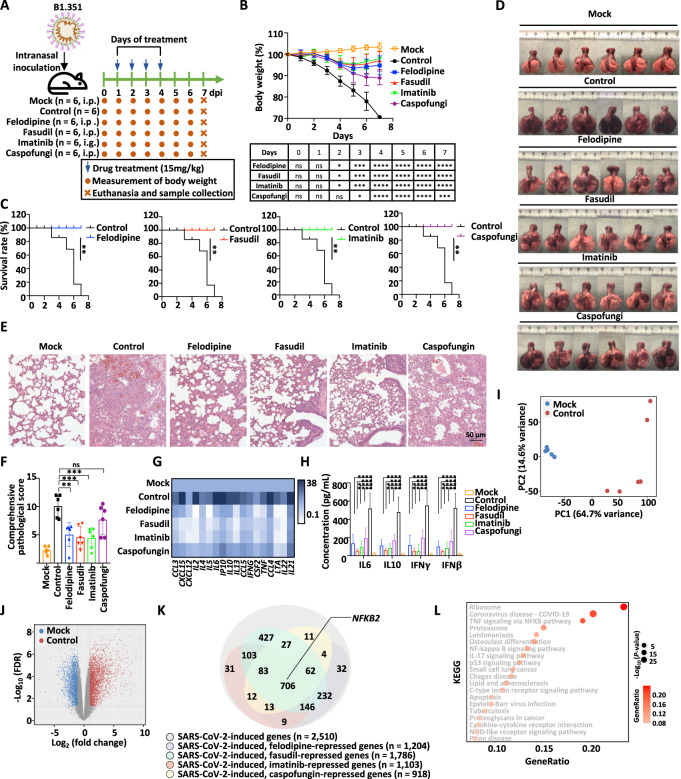
Felodipine, fasudil, imatinib, and caspofungin attenuate lethal inflammation, ameliorate severe pneumonia, and prevent mortality in SARS-CoV-2-infected Syrian hamsters. **A** Schematic representation of SARS-CoV-2 infection and drug treatment. Hamsters were intranasally inoculated with SARS-CoV-2 (1 × 10^4^ PFU) and then treated with or without felodipine (i.p.), fasudil (i.p.), imatinib (i.g.), or caspofungin (i.p.) on the indicated days (*n* = 6). Hamsters intranasally inoculated with SARS-CoV-2 without treatment were used as controls. Body weight was measured daily. Animals were euthanized to collect tissue samples at Day 7 post infection for virological and histological analysis. **B** The body weight of hamsters in each group from Day 0 to 7 post infection is shown. Significance was calculated using two-way ANOVA and is shown in the table (*P < 0.05, **P < 0.01, ***P < 0.001, ****P < 0.0001, ns: nonsignificant). **C** The survival curve for hamsters in each group from Day 0 to 7 post infection is shown. Significance was calculated using the log-rank (Mantel‒Cox) test (**P < 0.01). **D** Gross images of lung tissues collected from hamsters at Day 7 post infection are shown. **E** Representative images after H&E staining for lung lobe sections in hamsters at Day 7 post infection are shown. Scale bars, 50 μm. **F** Comprehensive pathological scores based on the severity and percentage of injured areas for each lung lobe for lung sections shown in Fig. S3A are shown. Significance was calculated using one-way ANOVA (*P < 0.05, **P < 0.01, ***P < 0.001, ****P < 0.0001, ns: nonsignificant). **G** The expression of inflammatory genes in lung tissues collected from hamsters at Day 7 post infection was detected by RT‒qPCR and represented by a heatmap. Data presented are the normalized value to the mock group after normalization to the expression of β-actin. The significance test is shown in Fig. S3B (*P < 0.05; **P < 0.01; ***P < 0.001). **H** The levels of cytokines, including IL6, IL10, IFNγ, and IFNβ in the lung tissues collected from hamsters at Day 7 post infection were measured by ELISA. Significance was calculated using one-way ANOVA (*P < 0.05, **P < 0.01, ***P < 0.001, ****P < 0.0001). **I** RNA was extracted from the lungs of hamsters as described in **A** at Day 7 post infection followed by RNA-seq analysis. The similarity in the gene expression profile for the mock and SARS-CoV-2-infected groups is shown by a PCA plot. **J** Genes regulated by SARS-CoV-2 infection are shown in a volcano plot. Blue and red dots represent down- and upregulated genes, respectively (FDR < 0.01, FC > 1.5). **K** Genes that were upregulated by SARS-CoV-2 infection as shown in **J** but repressed by felodipine, fasudil, imatinib, and caspofungin are shown in a Venn diagram. **L** KEGG analysis for those 706 genes that were upregulated by SARS-CoV-2 infection but commonly suppressed by felodipine, fasudil, imatinib, and caspofungin as described in **K** is shown

To determine whether the four drugs (felodipine, fasudil, imatinib, and caspofungin) screened by the SARS-CoV-2-S CAR-T-cell model could dampen inflammation to protect against SARS-CoV-2 infection and subsequent lethality, we treated SARS-CoV-2-infected Syrian hamsters with these drugs [54, 55]. Specifically, 6- to 8-week-old male Syrian hamsters were intranasally inoculated with SARS-CoV-2 (strain B1.351, 1 × 10^4^ plaque-forming units (PFUs)), which mimics the severe and lethal pneumonia caused by SARS-CoV-2 in COVID-19 patients (Fig. 5A). SARS-CoV-2-infected hamsters were treated with or without drugs (felodipine, i.p.; fasudil, i.p.; imatinib, i.g.; and caspofungin, i.p.) sequentially at Days 1, 2, 3, and 4 post infection (Fig. 5A). The untreated hamsters exhibited weight loss of up to 20% at Day 7 post infection (Fig. 5B). All four drugs rescued the loss of body weight from Day 2 post infection, and felodipine, fasudil, and imatinib exhibited a much better effect than caspofungin, which was consistent with the observation that the first three were much more potent in inhibiting cytokine release in vitro (Figs. 4E and 5B). Strikingly, all hamsters that received drug treatment survived at Day 7 post infection, while hamsters without treatment all died within 7 days after infection (Fig. 5C).

To evaluate the changes in virological, pathological, and immunological features responsive to treatment, tissues from organs in the respiratory tract, including the lung, turbinate, and trachea, were collected. Severe lung lesions, including consolidation and multifocal and diffuse hyperemia, were observed in the lungs of the hamsters without treatment, and these lesions were remarkably ameliorated in the groups that were treated with felodipine, fasudil, or imatinib (Fig. 5D). A comprehensive pathological score based on alveolar septum thickening and consolidation, hemorrhage, exudation, pulmonary edema and mucous, and recruitment and infiltration of inflammatory cells for lung lobes further confirmed the outstanding therapeutic effects of felodipine, fasudil, and imatinib (Figs. 5E, F, S7A and Table S1). Caspofungin displayed inadequate protection against SARS-CoV-2-induced lung injury when compared to the other three drugs (Figs. 5D–F, S7A and Table S1). Importantly, the protective effects of these drugs were shown to be associated with their suppression of the expression of inflammatory genes and the release of cytokines in the lung 7 days post infection (Figs. 5G, H and S7B). We also analyzed viral replication in organs, including the nasal turbinate, trachea, and lung, in the respiratory tract by examining the expression of SARS-CoV-2 open reading frame 1ab (ORF1ab) and nucleocapsid protein (NP) 7 days post infection. Our results indicated that SARS-CoV-2 RNA levels remained unchanged in the nasal turbinate, throat, and lung in response to drug treatment (Fig. S8A). Taken together, with these findings, we demonstrated that felodipine, fasudil, and imatinib were effective (and caspofungin to a lesser extent) therapeutic strategies against lethal inflammation in SARS-CoV-2-infected Syrian hamsters.

To further investigate the protective mechanism of these drugs in SARS-CoV-2-infected hamsters, we performed RNA-seq using RNA extracted from the lungs of Syrian hamsters in each group at 7 days post infection. Compared to mock hamsters, 2510 and 1947 genes were significantly up- and downregulated in SARS-CoV-2-infected hamsters, respectively (Fig. 5I, J). Among the 2510 genes that were upregulated by SARS-CoV-2 infection, 706 genes, including NF-κB2, were found to be commonly suppressed by felodipine, fasudil, imatinib, and caspofungin treatment (Fig. 5K). TNFα signaling via the NFκB pathway was one of the top three most enriched Kyoto Encyclopedia of Genes and Genomes (KEGG) pathways for these 706 genes (Fig. 5L). The suppression of NF-κB2 by felodipine, fasudil, imatinib, and caspofungin was confirmed by RT‒qPCR analysis (Fig. S8B).

### Discussion

**Fig. 6:**
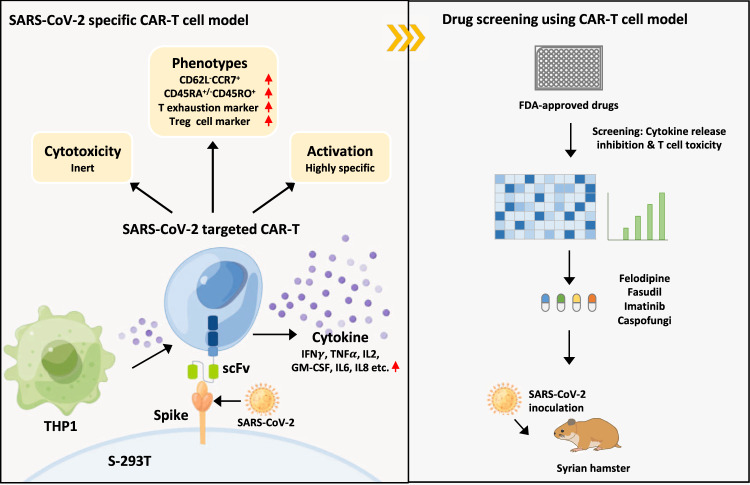
A SARS-CoV-2-specific CAR-T-cell model identifies felodipine, fasudil, imatinib, and caspofungin as effective treatments for SARS-CoV-2-infected Syrian hamsters. We demonstrated the feasibility of producing SARS-CoV-2-S CAR-T cells that have immunological profiles similar to those of T cells from COVID-19 patients in the presence of the S protein. This CAR-T-cell model can be used to screen drugs to suppress SARS-CoV-2-induced cytokine release. Among the drugs screened, felodipine, fasudil, imatinib, and caspofungin are effective in protecting against SARS-CoV-2-induced lethal inflammation in Syrian hamsters

Cryo-EM structure analysis indicated that the RBD domain of the spike protein of SARS-CoV-2 binds to the receptor ACE2 in host cells [56], allowing SARS-CoV-2 to enter cells for viral replication [57]. Since the RBD domain of the S protein is an immunodominant and highly specific target for antibodies [58], it has great potential to serve as a target for CAR engineering. Previously, we screened and obtained a murine neutralizing monoclonal antibody targeting the RBD of SARS-CoV-2 [17], of which scFv was used to generate the CAR-targeting S protein of SARS-CoV-2 in this study. T cells require both primary and costimulatory signals for optimal activation. In our SARS-CoV-2-S CAR-T-cell model, the primary antigen-specific signal is delivered by scFv antibody binding. The engagement of the costimulatory molecule CD28 mediates the second signal [59], which allows engineered T cells to be activated in an MHC-unrestricted mode and display function in an antigen-dependent manner (Fig. 6).

Recently, the nucleocapsid (N) protein of SARS-CoV-2 is reported to promote NLRP3 inflammasome activation [60]. It has also been reported that the N protein interacts with Smad3 to mediate intracellular Cl^−^ accumulation to trigger hyperinflammation [61]. However, the S protein of SARS-CoV-2 was demonstrated to be the key antigen to trigger innate and adaptive immune responses that lead to hyperinflammation [62–64]. In this study, we generated SARS-CoV-2-specific CAR-T cells and stimulated them with S proteins, which mimics the signatures of T-cell responses seen in COVID-19 patients, including distinct memory, exhausted, and regulatory T-cell phenotypes [22]. The alveolar space in the majority of patients with SARS-CoV-2 infection was persistently enriched in T cells and monocytes/macrophages. Alveolar macrophages can secrete T-cell chemoattractants [36]. Additionally, T cells produce IFNγ to induce inflammatory cytokine release from alveolar macrophages and further promote T-cell activation [36]. To better mimic the SARS-CoV-2-induced cytokine storm, we introduced THP1 cells into the coculture system with SARS-CoV-2-S CAR-T and S-293T cells. Similar to what is seen in COVID-19 patients, THP1 cells augmented cytokine release from CAR-T cells. IL8 was demonstrated to be the most secreted cytokine, which was again consistent with previous reports showing that it is one of the most significantly upregulated inflammatory genes in COVID-19 patients in single-cell RNA-seq analysis. Zaid, et al. also suggested that IL8 was 200 times more abundant than IL6 and TNFα in the lungs of patients with severe COVID-19 [65]. Based on these similarities, the SARS-CoV-2-S CAR-T-cell model might serve as a powerful tool to screen for drugs that can suppress SARS-CoV-2-induced cytokine storm and inflammation, the main cause of lethality in COVID-19. Different SARS-CoV-2 variants might cause varied degrees of cytokine storm and severities of COVID-19. For instance, the prototype virus [55] and early variants such as D614G [66], N501Y [67], and Delta [68] are likely to induce robust cytokine storm. In contrast, the Omicron variant usually induces a mild and transient cytokine storm [66, 69]. The mutated S protein from the SARS-CoV-2 Delta variant can also be recognized by the RBD antibody we used in our CAR-T model. Consistently, SARS-CoV-2-S CAR-T cells were activated in the presence of Delta-293T cells, producing significant amounts of inflammatory cytokines.

At present, the COVID-19 treatment armamentarium is largely represented by antiviral agents and immunotherapeutic agents that modulate the host immune response, including nonspecific and broad modulators, such as corticosteroids, or very targeted inhibitors, such as inhibiting one specific cytokine or kinase [70]. The tyrosine inhibitor imatinib, which was screened by our system, has already shown a beneficial effect compared with placebo in a clinical trial of 400 hospitalized patients with COVID-19 [71]. This study proves the feasibility of our CAR-T-cell model as an effective screening method. In our study, felodipine, fasudil, imatinib, and caspofungin were demonstrated to be effective in suppressing the release of cytokines from CAR-T cells, which was likely due to their ability to suppress the NF-κB pathway. Emerging evidence also suggests that these drugs might regulate the NF-κB signaling pathway. For example, it was demonstrated that the NF-κB pathway could be stimulated when L-type calcium channels are activated [72]. Felodipine has been reported to be a long-lasting L-type calcium channel repressor [73]. Felodipine has also been reported to attenuate vascular inflammation by inhibiting NF-κB activation in a rat model [74]. Fasudil hydrochloride is a selective Rho kinase ROCK inhibitor [41–43]. ROCK can promote the production of TNFα via NF-κB activation, and fasudil treatment inhibits the activation of NF-κB and production of proinflammatory cytokines in inflammatory disease and cancer [75, 76]. Imatinib potently inhibits several tyrosine kinases, including Bcr-Abl and the platelet-derived growth factor receptor (PDGFR) [43, 45], which were reported to induce the activation of the NF-κB pathway [77–79]. In CML patients, imatinib treatment can inhibit NF-κB expression and inflammatory cytokine production [80].

According to different treatment regimens, treatment with the drugs in our study could be labeled early intervention [12]. In a recent study, Marazzi, et al. revealed that the inhibition of topoisomerase 1 (Top1), a factor known to activate bacterial and viral infection-induced gene programs [81], suppressed the lethal inflammation induced by SARS-CoV-2 in vitro and in vivo [12]. Interestingly, early treatment with TOP1 inhibitor did not display a protective effect in mice. They believed that the inhibition of inflammation could be detrimental during the early phases of the infection [12]. However, we demonstrated that early intervention of inflammatory responses will be beneficial for host immunity, preventing SARS-CoV-2 infection-induced cytokine storm, disease progression, and subsequent multiple organ failure and death. On the basis of our results, we speculate that felodipine, fasudil, imatinib, and possibly caspofungin could be used as prophylactic treatments once patients are diagnosed with COVID-19. Especially for older people and people with medical conditions, the treatment of symptoms with these drugs at home or in clinics in the current stage might be helpful. Most importantly, these drugs are safe enough and have been regularly used for other diseases. They are also inexpensive and easily accessible for immediate use throughout the world.

In summary, the CAR-T-cell-based model may be applied for screening anti-inflammatory drugs for infectious disease-induced cytokine storm in a fast and high-throughput way. Recently, a global study led by the World Health Organization (WHO) tested new drugs, including imatinib, in hospitalized COVID-19 patients [82]. For our study, clinical trials are needed to evaluate the feasibility of repurposing the other three drugs, fasudil, felodipine, and caspofungin, for treating COVID-19 in humans.

## Methods

### Cell lines and cell culture

293 T and BEAS-2B cells were obtained from the Cell Bank of Chinese Academy of Sciences, Shanghai. Cells were maintained in high glucose Dulbecco’s modified Eagle’s medium (DMEM) (Biological Industries, Kibbutz Beit Haemek) supplemented with 10% heat-inactivated fetal bovine serum (FBS; Gibco, Grand Island). PBMCs were isolated from the whole blood of healthy volunteer donors using the Ficoll (GE Healthcare, Chicago) density gradient centrifugation method. For T-cell expansion, PBMCs were cultured and stimulated with anti-CD3 and anti-CD28 antibodies (Biolegend, San Diego) in X-VIVO medium (Lonza, Basel) supplemented with IL2 and IL15 (Sino, Beijing). Cells were cultured in a humidified incubator with 5% CO_2_ at 37 °C. This study was approved by the Ethics Committee of Xiamen University, and each donor signed an informed consent form before blood collection.

### Pseudovirus-based neutralization assay

Neutralizing activities of the anti-RBD antibody against SARS-CoV-2 wild-type and variants were quantified as previously described [17]. Diluted anti-RBD monoclonal antibody was mixed with VSV-SARS-CoV-2-Sdel18 pseudoviruses encoding the wild-type spike protein and its variants and then incubated at 37 °C for 1 h. The mixture was then transferred to BHK21 cells overexpressing human ACE2 (BHK21-hACE2) seeded in a 96-well microplate and incubated for 12 h. Fluorescence from the eGFP fused in viruses was captured by Opera Phenix (PerkinElmer, Waltham) and quantitatively analyzed by the Columbus system (PerkinElmer, Waltham). The percentage of the reduction of eGFP in each well compared with the control wells was calculated. IC_50_ values were determined by four-parameter logistic regression using GraphPad Prism (version 8.0.1).

### Plasmid construction and transfection

The spike gene of the Wuhan-Hu-1 strain (GenBank: MN908947) was synthesized and cloned into a pTT5-based vector or a pCAG-eGFP vector. VSV-SARS-CoV-2-Sdel18 (wild-type) was used as the template to generate different variants by site-directed mutagenesis and cloned into a pLV-mRuby-based vector. 293 T cells were seeded into 6-well culture plates and transiently transfected with these plasmids using Lipofectamine 3000 (Invitrogen, Carlsbad) following the manufacturer’s protocol for 24 hours to prepare S-293T, eGFP-S-293T, Omicron-293T, and Delta-293T cells. BEAS-2B cells stably expressing ACE2 (hACE2) were developed by the pTT5-based vector system (hACE2-BEAS-2B). S-hACE2-BEAS-2B was established based on infection with VSV-SARS-CoV-2-Sdel18 pseudovirus [17] and hACE2-BEAS-2B cells.

### Generation of SARS-CoV-2 S protein-targeted CAR-T cells

Monoclonal antibodies specifically targeting the spike (S) protein of SARS-CoV-2 were generated by immunizing BALB/c mice (Shanghai SLAC Laboratory Animal Center, Shanghai) using the RBD domain of the SARS-CoV-2 S protein (RBD, Fc Tag; Sino Biological Inc., Beijing). The second-generation SARS-CoV-2 S protein-targeted CAR construct (SARS-CoV-2-S CAR) was synthesized by subcloning the scFv (derived from SARS-CoV-2-specific monoclonal antibody) into the pCDH-based lentiviral plasmid encoding a signaling peptide (Sp1) of the IL2 receptor, a spacer (IgG1 Fc or hinge), a CD28 transmembrane domain, and the CD28 and CD3ζ intracellular signaling domains.

293 T cells were transfected with lentiviral and packaging vectors (CAR: psPAX: pMD2G = 4:3:1) using polyethylenimine (Mw 40000; Polysciences, Warrington) for 60 hours according to the manufacturer’s instructions before collecting supernatants, which were further concentrated 40-fold by using Amicon Ultra15 Centrifugal Filters (100 kDa; Millipore, Billerica). Concentrated lentiviruses were stored at –80 °C. Titrations were measured by the qPCR Lentivirus Titration (Titer) Kit (Applied Biological Materials, Crestwood Place Richmond).

Primary T cells were then subjected to lentivirus infection. To improve infection efficiency, T cells were spun at 800 × *g* for 50 mins at 32 °C after adding the lentiviral supernatant. Cells were monitored daily, and the culture medium was replaced every 2 to 3 days. T cells infected with control lentivirus (control T) were used as a negative control.

### Flow cytometry

The expression of the T-cell surface marker CD3 was detected using a FITC- or APC-conjugated mouse anti-human CD3 antibody (BD Biosciences, San Jose). The expression of the T-cell surface marker CD25 was detected using a FITC- or APC-conjugated mouse anti-human CD25 antibody (BD Biosciences, San Jose). The expression of the T-cell surface marker CD45RO was detected using a PE- or FITC-conjugated mouse anti-human CD45RO antibody (Biolegend, San Diego). The expression of the T-cell surface markers CD4, CD8, CD69, CCR7, CD62L, PD-1, TIM3, LAG3, FOXP3, and CD45RA was detected using a PE-conjugated mouse anti-human CD4 antibody, an APC-conjugated mouse anti-human CD8 antibody, a PE-conjugated mouse anti-human CD69 antibody, a FITC-conjugated anti-human CCR7 antibody, a PE-conjugated anti-human CD62L antibody, a FITC-conjugated mouse anti-human CD279 (PD-1) antibody, an APC-conjugated mouse anti-human CD366 (TIM-3) antibody, a PE-conjugated mouse anti-human CD223 (LAG-3) antibody, an Alexa Fluor 488 anti-human FOXP3 antibody and an APC-conjugated mouse anti-human CD45RA antibody (BD Biosciences, San Jose). CAR expression in 293 T cells was examined based on the eGFP fused with the S protein. The infection efficiency of Fc-SARS-CoV-2-S CAR in 293 T cells or T cells was examined by using an APC-conjugated anti-human IgG-Fc antibody (Abcam, Cambridge). For FOXP3 detection, T cells were fixed and permeabilized for 40 minutes before staining with anti-FOXP3 antibody. For human ACE2 detection, BEAS-2B or hACE2-BEAS-2B cells were examined by using an Alexa Fluor® 647 anti-human ACE2 antibody (Biolegend, San Diego). All fluorescence was assessed using an Attune NxT Flow Cytometer (Thermo Fisher Scientific, Waltham), and the data were analyzed with FlowJo vX.0.7 (BD Biosciences, San Jose).

### Cytokine detection

Control T or SARS-CoV-2-S CAR-T cells were incubated with 293 T or S-293T cells at different ratios (T cells: 293 T cells = 1:1, 3:1, 6:1 or 10:1) in a 48-well plate (Corning Incorporated, Corning) for 72 hours, and supernatants were collected to determine the presence of IFNγ, TNFα, IL2, perforin, GM-CSF, granzyme B, IL6, and IL10 using the Human IFNγ Enzyme-linked immunosorbent assay (ELISA) Kit, Human TNFα ELISA Kit, Human IL2 ELISA Kit, Human Perforin ELISA Kit, Human GM-CSF ELISA Kit, Human Granzyme B ELISA Kit, Human IL6 ELISA Kit, and Human IL10 ELISA Kit, respectively, following the manufacturer’s instructions (Dakewe Biotech, Beijing). The secretion of multiple cytokines was measured simultaneously by a multiplex bead array using the Cytokine & Chemokine 34-Plex Human ProcartaPlex™ Panel (Invitrogen, EPX340-12167-901) according to the manufacturer’s instructions. For cytokine detection in hamsters, the lung tissues of hamsters in each group were cleaved into small pieces, ground and suspended as cell supernatants to determine the presence of IL6, IL10, IFNγ, and IFNβ using the Hamster IL6 ELISA Kit, Hamster IL10 ELISA Kit, Hamster IFNγ ELISA Kit, and Hamster IFNβ ELISA Kit (Cusabio, USA), respectively.

### Cell proliferation assay

Control T or SARS-CoV-2-S CAR-T cells were incubated with 293 T, S-293T or Omicron-293T cells in culture medium without adding proliferative cytokines in a 6-well plate (T cells: 293 T cells = 2:1) (Corning Incorporated, Corning). After incubation, T cells in suspension were then separated from adherent 293 T, S-293T, or Omicron-293T cells; collected; and stained with trypan blue on Days 2, 4, 6, 8, 10, or 12. The number of viable T cells was counted by using a hemocytometer (Paul Marienfeld, Lauda-Koenigshofen).

### Cytotoxicity assay

The cytotoxicity assay was performed using an xCELLigence real-time cell analyzer (RTCA) System (ACEA Biosciences, San Diego). Impedance-based RTCA was used for label-free and real-time monitoring of cytolysis activity. The cell index (CI) based on the measured cell-electrode impedance was used to measure cell viability. Cytotoxicity was calculated via the following formula: ((CI (target cells only) – CI (target cells + T cells))/CI target cells only) × 100%. S-293T cells were seeded at a density of 1 × 10^4^ cells per well and grown for 24 hours. Control T or SARS-CoV-2-S CAR-T cells with or without drug treatment were then added to the RTCA unit at different ratios (T cells: S-293T cells = 1:1, 3:1, 6:1 or 10:1 or 5:1). The impedance signals were recorded for 24–96 hours at 5-min intervals.

For GFP fluorescence detection, eGFP-S-293T cells were seeded at a density of 2 × 10^4^ cells per well. CTL T or SARS-CoV-2-S CAR-T cells were then added to eGFP-S-293T cells at different ratios (T cells: eGFP-S-293T cells = 1:1, 3:1, 6:1 or 10:1). Fluorescence images were obtained by Operetta CLS equipment (Perkin-Elmer, Waltham) at 96 hours. For quantitative determination, fluorescence images were analyzed, and the numbers of GFP-positive cells were counted by a Columbus system (Perkin-Elmer, Waltham).

### RNA isolation and RT‒qPCR

Control T or SARS-CoV-2-S CAR-T cells were incubated with S-293T cells at a ratio of 3:1 for two days, and T cells in suspension were separated from adherent S-293T cells. Both types of cells were collected and centrifuged at 1000 rpm for 5 min before RNA extraction followed by RT‒qPCR analysis. Dead T cells were removed using the Dead Cell Removal Kit (Miltenyi Biotec, Auburn). Total RNA was isolated using the Eastep Super Total RNA Extraction Kit (Promega, Madison) following the manufacturer’s protocol. First-strand complementary DNA (cDNA) synthesis from total RNA was carried out using the GoScript Reverse Transcription System (Promega, Madison), followed by quantitative PCR (qPCR) using an AriaMx Real-Time PCR machine (Agilent Technologies, Santa Clara). For cytokine mRNA detection in hamsters, the lung tissues were cleaved into small pieces and soaked in RNAlater (#AM7021, Invitrogen). Total RNA in lysed lung tissues was extracted with an RNeasy Mini kit (#74106, Qiagen) and reverse-transcribed to cDNA with a Fast-King Strand cDNA Synthesis Kit (#FP313, TIANGEN, Beijing).

### Immunoblotting analysis

Immunoblotting was performed as described previously [83]. Anti-p100/52 (4882) and anti-β-Actin rabbit mAb (High Dilution) (AC026, Abclonal, Wuhan) as well as HRP-labeled secondary antibodies (Cell Signaling Technology, Massachusetts) were used in this study.

### Bulk RNA sequencing analysis

Public bulk RNA sequencing datasets were downloaded from the Gene Expression Omnibus (https://www.ncbi.nlm.nih.gov/geo/). The COVID-19 dataset used in this study was NO. GSE166992. This dataset contained PBMCs from three healthy controls and six COVID-19 patients. Seurat 4.1.0 was used to process and analyze the COVID-19 dataset. We removed low-quality cells by selecting feature numbers between 200 and 4000. Moreover, we removed cells with a mitochondrial percentage >15% and gene number between 250 and 4000. The bulk RNA-seq datasets were normalized and variance stabilized by the “sctransform V2” function from the official website of Seutat (https://satijalab.org/).

### SARS-CoV-2-S CAR-T-cell-based high-throughput screening of drugs that can suppress SARS-CoV-2-induced inflammation

S-293T cells were seeded into 96-well culture plates (20,000 cells/well) and incubated at 37 °C and 5% CO2 overnight (~12 h). SARS-CoV-2-S CAR-T (10:1 to S-293T) and THP1 cells (10:1 to S-293T) were added to S-293T cells to produce multiple inflammatory cytokines. Drugs from the FDA-approved drug library (MCE, HY-LD-000001083) were then added to the wells at a concentration of 10 μM. After 72 h of coculture, the cell supernatant was collected for IL8 and IFNγ detection by ELISA. Drugs screened to inhibit the secretion of IL8 and IFNγ were further tested for toxicity to T cells. In brief, drugs were cocultured with T cells at a concentration of 10 μM. After incubation for 72 h, T cells were stained with trypan blue and counted by using a hemocytometer to calculate the toxicity of drugs to T cells.

### RNA-seq analysis

SARS-CoV-2-S CAR-T cells and THP1 cells were incubated with or without S-293T cells at a ratio of 3:1 for two days, and CAR-T cells were then separated from adherent cells and spun down at 1000 rpm for 5 min. Dead cells were removed using the Dead Cell Removal Kit (Miltenyi Biotec, Auburn) before RNA extraction and RNA-seq analysis. Alternatively, suspension CAR-T cells were incubated with S-293T cells at a ratio of 3:1 and treated with or without drugs at a concentration of 10 μM for 48 hours followed by RNA-seq analysis. Three biological replicates were performed, and RNA was pooled for sequencing. Lung tissues collected from treated or untreated hamsters (*n* = 6) were also subjected to RNA extraction and RNA-seq analysis. Total RNA was isolated using the RNeasy Mini Kit (Qiagen) following the manufacturer’s protocol. DNase I (Sigma‒Aldrich) in the column digestion was included to ensure RNA quality. RNA library preparation was performed by using the NEBNext® Ultra™ Directional RNA Library Prep Kit (Illumina, San Diego). Paired-end sequencing was performed with Illumina HiSeq 3000. Sequencing reads were aligned to the hg19 RefSeq database by using Tophat (http://ccb.jhu.edu/software/tophat/index.shtml). Cuffdiff was used to quantify the expression of RefSeq annotated genes with the options –M (reads aligned to repetitive regions were masked) and –u (multiple aligned reads were corrected using the ‘rescue method’). Coding genes with fragments per kilobase per million (FPKM) mapped reads larger than 0.5 were included in our analysis. Up- and downregulated genes were determined by the fold change in gene FPKM. The FPKM of a gene was calculated as the mapped reads on exons divided by the exonic length and the total number of mapped reads. Hallmark and KEGG pathway analyses were performed using the Metascape web server.

### Experimental animal and biosafety

Syrian hamsters (LVG strain) were raised in specific pathogen-free animal feeding facilities. All animal experiments were approved by the Medical Ethics Committee (SUCM2021-112). All experiments with infectious SARS-CoV-2 were performed in biosafety level 3 (BSL-3) and animal biosafety level 3 (ABSL-3) facilities affiliated with the State Key Laboratory of Emerging Infectious Diseases, The University of Hong Kong. Our staff wear powered air-purifying respirators that filter the air and disposable coveralls when they culture the virus and handle animals that are in isolators. The researchers are disinfected before they leave the room and then shower on exiting the facility. All facilities, procedures, training records, safety drills, and inventory records are subject to periodic inspections and ongoing oversight by institutional biosafety officers who consult frequently with facility managers.

### Preparation of virus stock

The SARS-CoV-2 B.1.351 mutation virus strain AP-100 (hCoV19/China/AP100/2021; GISAID accession number: EPI_ISL_2779638) was passaged in Vero cells (#CCL-81, ATCC). Viral stocks were prepared in Vero cells with DMEM containing 2% FBS, 5 µg/mL TPCK-trypsin, penicillin‒streptomycin and 30 mmol/L MgCl2 (#11995, #10270106, #T1426 and #15140-122; purchased from Gibco, Sigma‒Aldrich and Invitrogen). Viruses were harvested and stored in an ultralow temperature refrigerator. The titers were determined by means of a plaque assay in Vero cells.

### Virus inoculation and treatment

The hamsters were anesthetized by isoflurane (#R510-22, RWD Life Science) and nasally inoculated with a 1 × 10^4^ PFU dose of SARS-CoV-2 diluted in 200 µL PBS (#10010031, Gibco). After SARS-CoV-2 infection, these hamsters were observed daily for illness symptoms, including weakness, piloerection, hunched back, and abdominal respiration. For treatment, fasudil (HY-10341, MCE), caspofungin (HY-17006, MCE) or imatinib (HY-50948, MCE) was dissolved in PBS, and felodipine predissolved in DMSO was then diluted with sesame oil (#S25527, Shanghai Yuanye). On the first four days, hamsters were treated with isoflurane daily, followed by intraperitoneal injection (i.p.) of fasudil, felodipine, or caspofungin or intragastric injection (i.g.) of imatinib at a dose of 15 mg/kg. The body weights of these hamsters were measured daily by an electronic balance. Hamsters were euthanized and sacrificed at the indicated time points for the determination of viral load in respiratory tract organs and the analysis of pathogenesis in lung lobes.

### Detection of viral RNA

Viral RNA was extracted by using a QIAamp Viral RNA Mini kit (#52906, Qiagen) according to the manufacturer’s instructions. RT‒qPCR was conducted by using the SLAN-96S Real-Time System (Hongshi, Shanghai, China) with a SARS-CoV-2 RT‒PCR Kit from Wantai (Beijing, China). Relative viral RNA levels of the SARS-CoV-2 ORF1ab gene and NP gene were determined using the primer pairs and probes shown in the kit instructions. Viral RNA copies were expressed on a log_10_ scale after normalization to the standard curve obtained by using tenfold dilutions of a SARS-CoV-2 stock.

### Histopathological studies

For pathological analysis, lung tissues were fixed in formalin for more than 48 hours, dehydrated and then embedded in paraffin wax. The wax block of lung tissues was cut into 4 μm sections for hematoxylin and eosin (H&E) staining. H&E staining was employed for the analysis of general lung pathogenic lesions, including pulmonary edema, consolidation and inflammation. The standards for the pathological score of lung tissues in this study were derived from our previous study in a hamster model. Comprehensive pathological scoring of lung sections was performed according to the degree of lung lesions, including alveolar septum hyperplasia, the consolidation and impairment of alveolar structure, fluid exudation, mucus suppository, thrombus, inflammation recruitment and the infiltration of immune cells in each lung lobe. For each hamster, three or four lung lobes were employed for the evaluation of the comprehensive pathological score. In brief, the H&E staining results of each lung lobe were analyzed to determine the severity of pathological changes. The pathological score includes a) alveolar septum thickening and consolidation; b) hemorrhage, exudation, pulmonary edema and mucous; and c) the recruitment and infiltration of inflammatory immune cells. For each issue, the score was related to the severity: 0 indicated no pathological change, 1 indicated moderate pathological change, 2 indicated mild pathological change, 3 indicated severe pathological change, and 4 indicated very severe pathological change. In conclusion, the scores of these three issues were added as the comprehensive pathological score of a lung lobe, and the average comprehensive pathological score of the lobes indicated the severity of lung pathogenesis in an evaluated hamster. The images of whole lung lobes were screened by a high-throughput screening microscope system (EVOS M7000, Invitrogen of Thermo Fisher Scientific).

### Statistical analysis

Comparison of two groups or data points was performed using a two-tailed *t* test. Statistical analyses of body weight of hamsters between multiple groups were performed using one-way analysis of variance (ANOVA) with Holm-Šídák’s multiple comparisons test or two-way ANOVA with Dunnett’s multiple comparisons test as needed. Survival curves were constructed according to the Kaplan‒Meier method and compared using the log-rank (Mantel‒Cox) test. Statistical significance was set at *P* < 0.05. Statistical analysis was performed using GraphPad Prism8 software (GraphPad Software Inc.).

## Supplementary information

Supplementary Figures

Table S1

Supplementary figure legends
