# Supplementary material for: A SARS-CoV-2-specific CAR-T-cell model identifies felodipine, fasudil, imatinib, and caspofungin as potential treatments for lethal COVID-19
Source: Cell Mol Immunol. 2023 Mar 2;20(4):351–64. doi: 10.1038/s41423-023-00985-3 (PMC9979130; doi:10.1038/s41423-023-00985-3)
Supplement: Supplementary file 1 — Supplementary Figures [file 41423_2023_985_MOESM1_ESM.pdf]

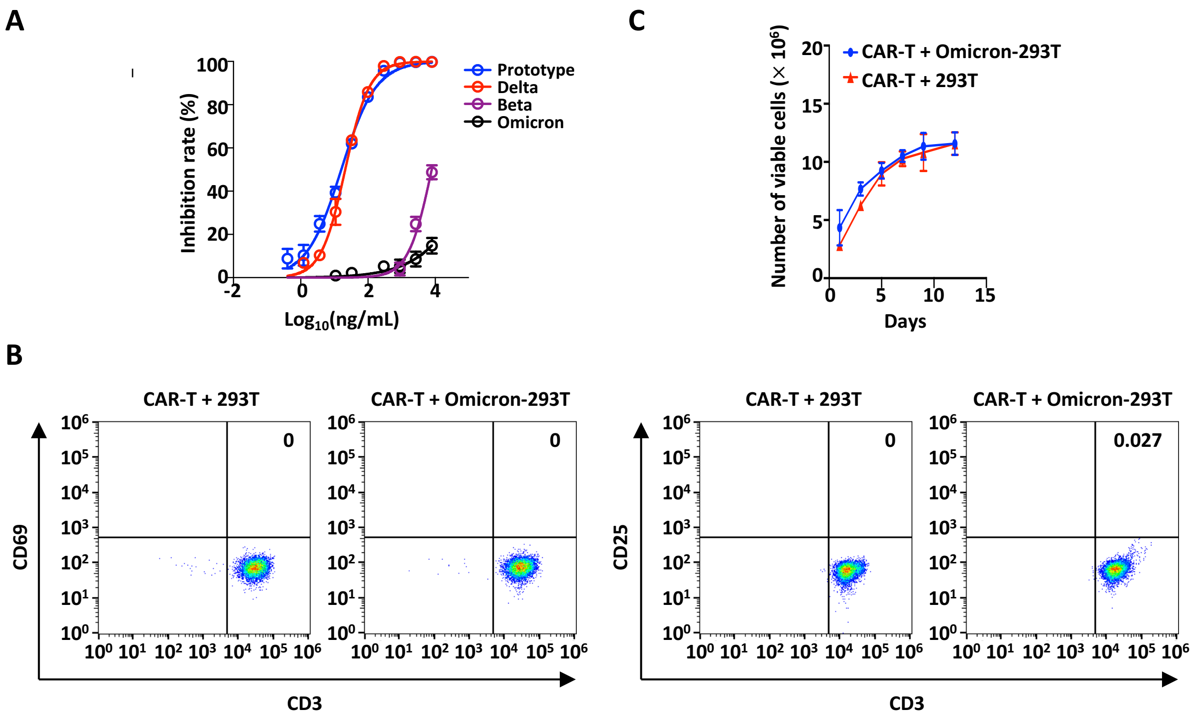
A
Inhibition rate (%)
Log10(ng/mL)
Prototype
Delta
Beta
Omicron
C
Number of viable cells (× 10⁶)
Days
CAR-T + Omicron-293T
CAR-T + 293T
B
CAR-T + 293T
CAR-T + Omicron-293T
CD69
CD25
CD3
0
0
0
0.027

Figure S1

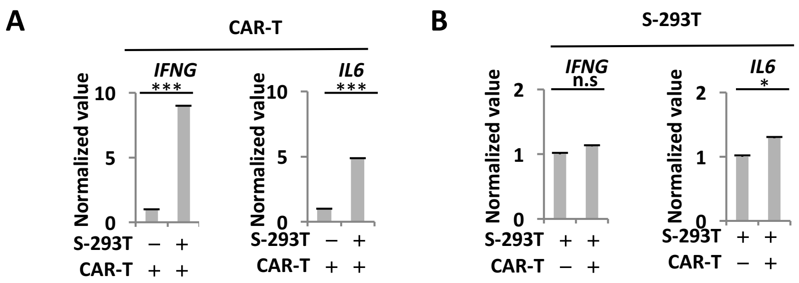
A
CAR-T
IFNG
***
IL6
***
Normalized value
10
5
0
S-293T – +
CAR-T + +
B
S-293T
IFNG
n.s
IL6
*
2
1
0
S-293T + +
CAR-T – +

**Figure S2**

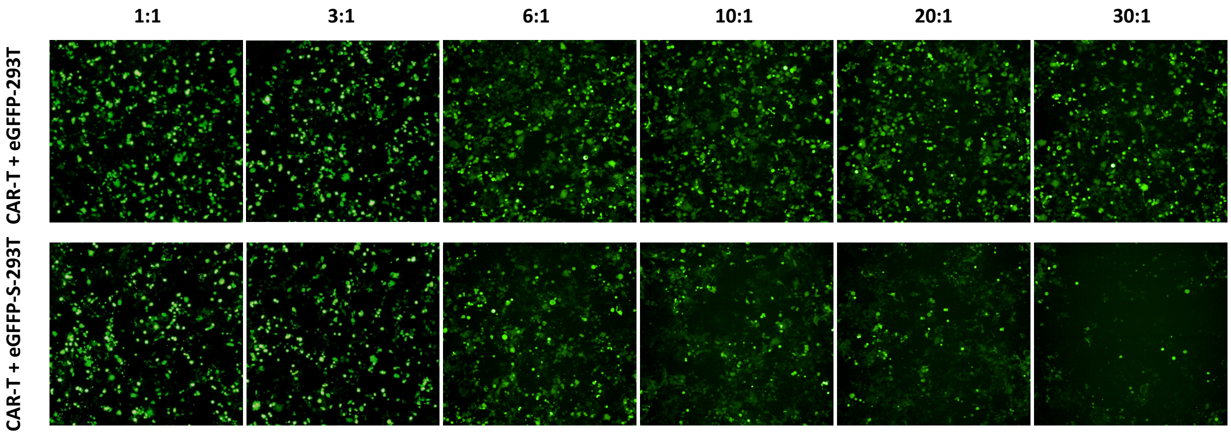


Figure S3

**A**

| | | CAR-T + 293T: + / CAR-T + S-293T: – | CAR-T + 293T: – / CAR-T + S-293T: + | |
|---|---|---|---|---|
| Proliferation | *IL2RA* | 1.00, ±0.00 | 1.51, ±0.02 | ** |
| | *IL2* | 1.00, ±0.00 | 1.90, ±0.31 | ** |
| Activation | *AP1* | 1.00, ±0.00 | 3.49, ±0.05 | *** |
| | *NFAT1* | 1.00, ±0.00 | 1.21, ±0.04 | * |
| | *RELA* | 1.00, ±0.00 | 2.31, ±0.06 | ** |
| | *RELB* | 1.00, ±0.00 | 2.27, ±0.17 | ** |
| | *VCAM1* | 1.00, ±0.00 | 9.23, ±0.50 | *** |
| IFNγ response | *IFNG* | 1.00, ±0.00 | 1.95, ±0.07 | * |
| | *CXCL10* | 1.00, ±0.00 | 4.40, ±0.19 | *** |
| | *CXCL11* | 1.00, ±0.00 | 5.08, ±0.53 | *** |
| Cytotoxicity | *FASLG* | 1.00, ±0.00 | 3.74, ±0.62 | *** |

Mean, ± SD

**B**

| | | CAR-T + 293T: + / CAR-T + S-293T: – | CAR-T + 293T: – / CAR-T + S-293T: + | |
|---|---|---|---|---|
| Naïve-associated genes | *ACTN1* | 1.00, ±0.00 | 0.78, ±0.07 | * |
| | *FOXP1* | 1.00, ±0.00 | 0.72, ±0.06 | * |
| | *TAF4B* | 1.00, ±0.00 | 0.63, ±0.29 | * |
| | *IL6ST* | 1.00, ±0.00 | 0.60, ±0.05 | ** |
| | *TCF7* | 1.00, ±0.00 | 0.57, ±0.01 | *** |
| | *CD62L* | 1.00, ±0.00 | 0.37, ±0.04 | *** |
| Effector-associated genes | *PRDM1* | 1.00, ±0.00 | 1.65, ±0.01 | ** |
| | *GZMA* | 1.00, ±0.00 | 2.17, ±0.07 | ** |
| | *TBX21* | 1.00, ±0.00 | 3.82, ±0.24 | *** |
| | *EOMES* | 1.00, ±0.00 | 5.45, ±0.19 | *** |
| | *KLRG1* | 1.00, ±0.00 | 5.32, ±0.04 | *** |
| | *PLEK* | 1.00, ±0.00 | 7.62, ±0.14 | *** |

Mean, ± SD

**C**

| | | CAR-T + 293T: + / CAR-T + S-293T: – | CAR-T + 293T: – / CAR-T + S-293T: + | |
|---|---|---|---|---|
| Exhaustion TF | *IRF4* | 1.00, ±0.00 | 1.81, ±0.05 | ** |
| | *BATF* | 1.00, ±0.00 | 1.50, ±0.12 | ** |
| Checkpoints | *BTLA* | 1.00, ±0.00 | 1.27, ±0.09 | * |
| | *LAG3* | 1.00, ±0.00 | 2.47, ±0.07 | *** |
| | *CTLA4* | 1.00, ±0.00 | 1.28, ±0.03 | * |
| | *TIM3* | 1.00, ±0.00 | 1.44, ±0.07 | ** |
| | *PD1* | 1.00, ±0.00 | 1.18, ±0.15 | * |

Mean, ± SD

**Figure S4**

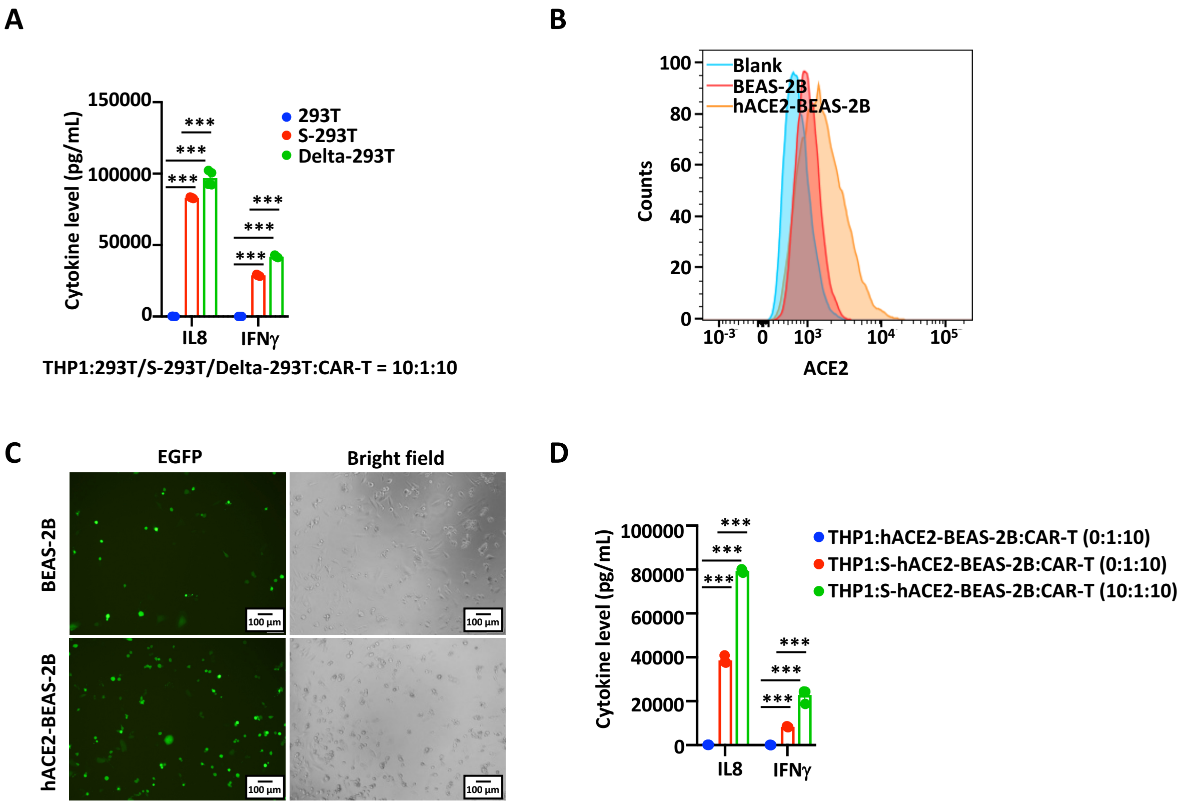
A
150000
100000
50000
0
Cytokine level (pg/mL)
***
293T
S-293T
Delta-293T
IL8
IFNγ
THP1:293T/S-293T/Delta-293T:CAR-T = 10:1:10
B
100
80
60
40
20
0
Counts
Blank
BEAS-2B
hACE2-BEAS-2B
10-3
0
103
104
105
ACE2
C
EGFP
Bright field
BEAS-2B
hACE2-BEAS-2B
100 µm
D
100000
80000
60000
40000
20000
0
Cytokine level (pg/mL)
THP1:hACE2-BEAS-2B:CAR-T (0:1:10)
THP1:S-hACE2-BEAS-2B:CAR-T (0:1:10)
THP1:S-hACE2-BEAS-2B:CAR-T (10:1:10)
IL8
IFNγ

Figure S5

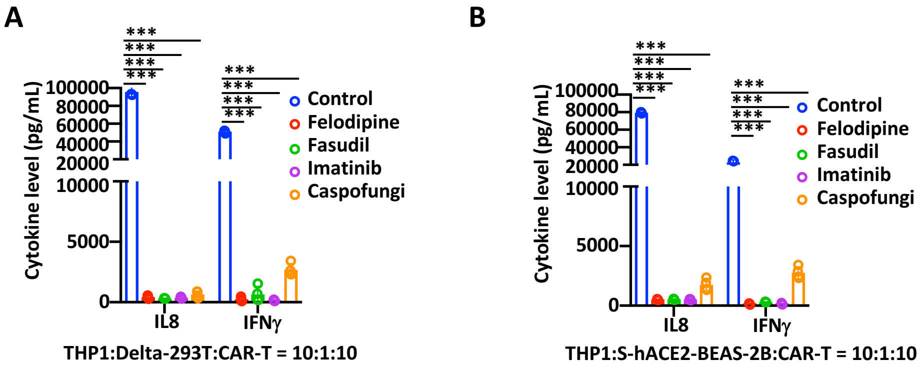
A
B
***
Cytokine level (pg/mL)
100000
80000
60000
40000
20000
10000
5000
0
IL8
IFNγ
Control
Felodipine
Fasudil
Imatinib
Caspofungi
THP1:Delta-293T:CAR-T = 10:1:10
THP1:S-hACE2-BEAS-2B:CAR-T = 10:1:10

**Figure S6**

**A**

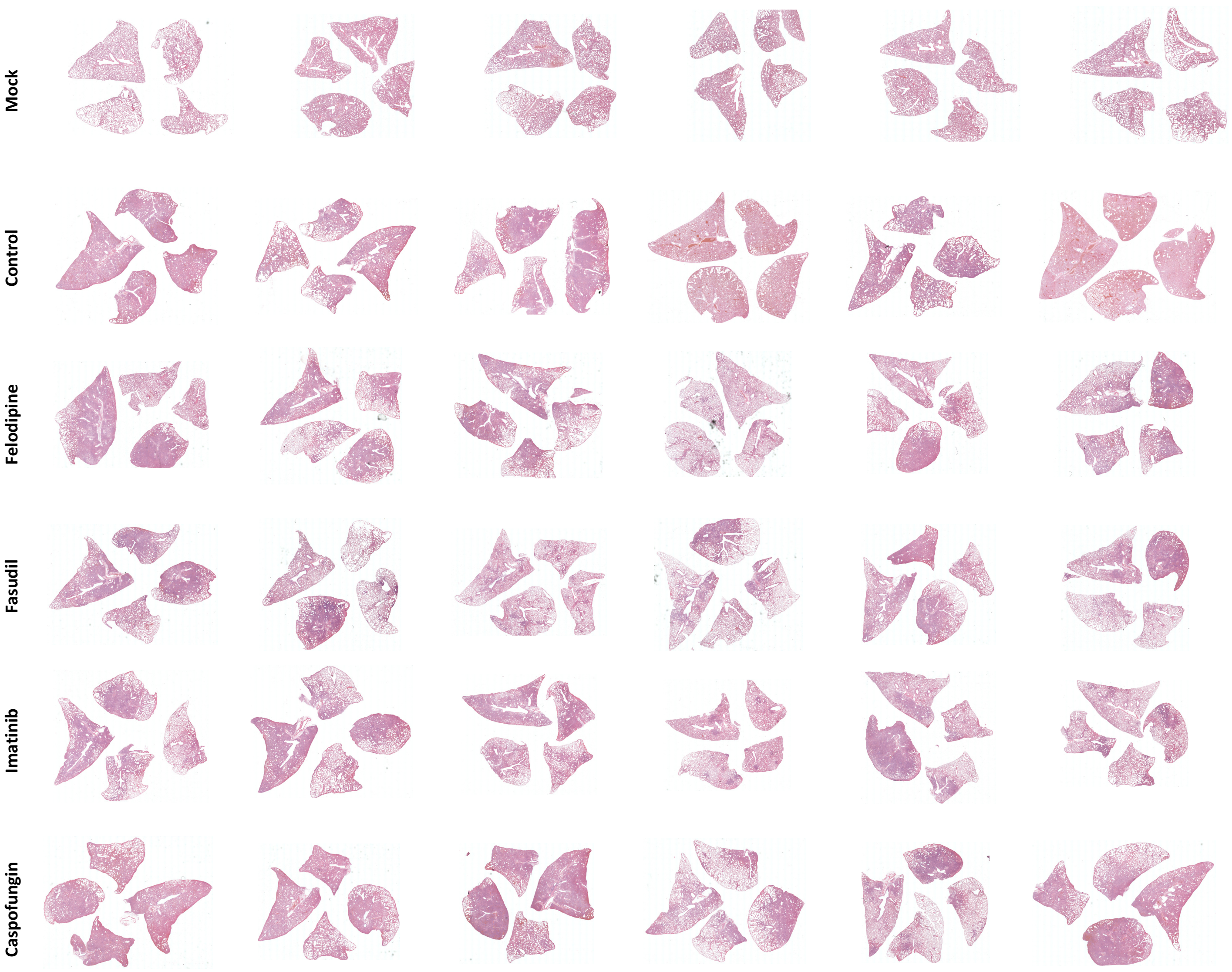


**B**

| CCL3 | CXCL15 | CXCL12 | IL2 | IL4 | IL5 | IL6 | IP10 | IL10 | IL13 | CCL5 | IFNG | CSF2 | TNF | CCL4 | LTA | IL22 | IL21 |
|---|---|---|---|---|---|---|---|---|---|---|---|---|---|---|---|---|---|
| 1.00,±0.00 | 1.00,±0.00 | 1.00,±0.00 | 1.00,±0.00 | 1.00,±0.00 | 1.00,±0.00 | 1.00,±0.00 | 1.00,±0.00 | 1.00,±0.00 | 1.00,±0.00 | 1.00,±0.00 | 1.00,±0.00 | 1.00,±0.00 | 1.00,±0.00 | 1.00,±0.00 | 1.00,±0.00 | 1.00,±0.00 | 1.00,±0.00 |
| 21.67,±2.80 | 95.1,±1.11 | 1.92,±1.11 | 37.68,±7.36 | 16.55,±4.11 | 19.50,±10.93 | 69.16,±9.93 | 50.65,±10.66 | 31.87,±9.98 | 31.48,±2.70 | 24.08,±3.99 | 18.81,±5.30 | 7.72,±0.40 | 23.63,±1.72 | 51.12,±2.97 | 24.10,±3.35 | 14.18,±2.87 | 62.46,±6.45 |
| 4.21,±0.71 | 1.95,±0.04 | 0.65,±0.06 | 0.11,±0.02 | 1.32,±0.90 | 0.14,±0.05 | 1.21,±0.15 | 11.87,±0.95 | 1.52,±0.16 | 0.43,±0.01 | 5.08,±0.74 | 1.77,±0.16 | 0.30,±0.01 | 0.47,±0.14 | 9.43,±0.82 | 0.19,±0.04 | 0.17,±0.02 | 15.70,±0.29 |
| 5.09,±2.33 | 0.45,±0.02 | 0.37,±0.00 | 0.27,±0.04 | 1.36,±0.28 | 0.32,±0.20 | 0.54,±0.12 | 12.90,±10.73 | 2.32,±0.27 | 1.16,±0.33 | 7.10,±0.22 | 1.74,±0.18 | 0.67,±0.05 | 0.63,±0.03 | 9.46,±0.87 | 0.40,±0.07 | 0.41,±0.09 | 18.16,±1.57 |
| 0.78,±0.05 | 0.52,±0.03 | 0.28,±0.04 | 0.26,±0.09 | 0.78,±0.02 | 0.46,±0.05 | 0.83,±0.12 | 1.50,±0.22 | 0.74,±0.06 | 0.39,±0.11 | 4.49,±0.51 | 1.22,±0.16 | 0.46,±0.03 | 0.68,±0.03 | 2.01,±0.18 | 0.28,±0.03 | 0.19,±0.01 | 5.53,±1.77 |
| 4.07,±0.22 | 1.93,±0.21 | 0.43,±0.19 | 0.15,±0.04 | 0.97,±0.45 | 0.32,±0.05 | 3.22,±0.70 | 14.36,±3.21 | 1.82,±0.08 | 0.58,±0.14 | 6.51,±0.09 | 1.69,±0.20 | 0.71,±0.06 | 0.47,±0.03 | 7.43,±0.79 | 0.34,±0.05 | 0.22,±0.02 | 17.71,±4.01 |

1 2 3 4 5

| CCL3 | CXCL15 | CXCL12 | IL2 | IL4 | IL5 | IL6 | IP10 | IL10 | IL13 | CCL5 | IFNG | CSF2 | TNF | CCL4 | LTA | IL22 | IL21 | |
|---|---|---|---|---|---|---|---|---|---|---|---|---|---|---|---|---|---|---|
| 0.0002 | 0.0001 | 0.2303 | 0.0010 | 0.0028 | 0.0427 | 0.0003 | 0.0013 | 0.0059 | 0.0000 | 0.0006 | 0.0043 | 0.0000 | 0.0000 | 0.0000 | 0.0003 | 0.0014 | 0.0001 | 1 |
| 0.0016 | 0.0006 | 0.0420 | 0.0007 | 0.5886 | 0.0003 | 0.1356 | 0.0000 | 0.0099 | 0.0000 | 0.0007 | 0.0015 | 0.0000 | 0.0027 | 0.0001 | 0.0012 | 0.0003 | 0.0000 | 2 |
| 0.0928 | 0.0014 | 0.0049 | 0.0016 | 0.2011 | 0.0073 | 0.0115 | 0.1275 | 0.0015 | 0.4457 | 0.0000 | 0.0026 | 0.0016 | 0.0000 | 0.0001 | 0.0046 | 0.0026 | 0.0001 | 3 |
| 0.1051 | 0.0142 | 0.0032 | 0.0022 | 0.2679 | 0.0021 | 0.1776 | 0.1052 | 0.0253 | 0.0007 | 0.0004 | 0.0981 | 0.0001 | 0.0001 | 0.0010 | 0.0019 | 0.0003 | 0.0129 | 4 |
| 0.0000 | 0.0003 | 0.0213 | 0.0009 | 0.9206 | 0.0008 | 0.0057 | 0.0020 | 0.0006 | 0.0077 | 0.0000 | 0.0048 | 0.0037 | 0.0000 | 0.0002 | 0.0028 | 0.0004 | 0.0020 | 5 |

**Figure S7**

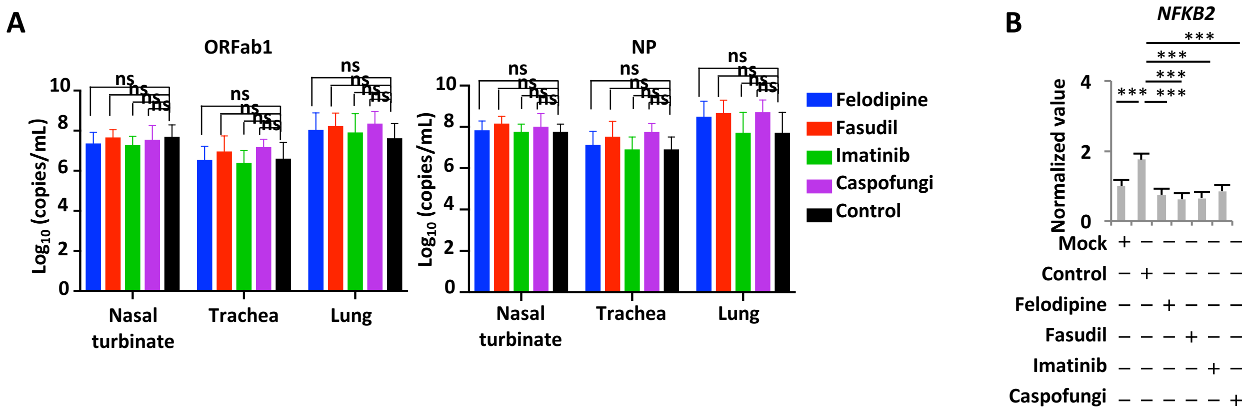
A
ORFab1
NP
Log10 (copies/mL)
10
8
6
4
2
0
ns
Nasal turbinate
Trachea
Lung
Felodipine
Fasudil
Imatinib
Caspofungi
Control
B
NFKB2
Normalized value
***
Mock
Control
Felodipine
Fasudil
Imatinib
Caspofungi

Figure S8
