# Supplementary material for: A SARS-CoV-2-specific CAR-T-cell model identifies felodipine, fasudil, imatinib, and caspofungin as potential treatments for lethal COVID-19
Source: Cell Mol Immunol. 2023 Mar 2;20(4):351–64. doi: 10.1038/s41423-023-00985-3 (PMC9979130; doi:10.1038/s41423-023-00985-3)
Supplement: Supplementary file 2 — Table S1 [file 41423_2023_985_MOESM2_ESM.pdf]

Table S1. The comprehensive pathological score for the lung lobes in hamsters treated with or without drugs

Group	Identifier	Pathological lesions			Comprehensive pathological score for lung lobes	Comprehensive pathological score for hamster
		Alveolar septum hyperplasia and consolidation	Pulmonary edema, hemorrhage and mucus suppository	Recruitment and infiltration of inflammatory cells		
Fasudil	C779	3+3+2+1	3+3+3+1	3+3+3+0	9+9+8+2	7
	C780	3+1+0+0	3+1+1+1	3+1+0+0	9+3+1+1	3.5
	C781	1+0+0+0	1+1+1+1	1+1+1+1	3+2+2+2	2.25
	C782	2+1+1+0	2+1+1+1	3+1+1+0	7+3+3+1	3.5
	C783	4+2+1+1	3+3+2+1	4+3+2+1	11+8+5+3	6.75
	C784	4+1+0+0	3+2+1+1	4+2+0+0	11+5+1+1	4.5
Caspofungi	C785	4+3+3+2	4+4+4+3	4+4+4+3	12+11+11+8	10.5
	C786	3+3+2+1	4+3+3+1	4+3+3+1	11+9+8+3	7.75
	C787	4+4+3+1	4+4+4+1	4+4+4+2	12+12+11+4	9.75
	C788	3+1+1+1	3+2+1+1	3+1+1+1	9+4+3+3	4.5
	C789	3+1+0+0	3+1+1+1	4+2+1+1	10+4+2+2	4.5
	C790	4+4+3+2	4+3+3+2	4+3+3+2	12+10+9+6	9.25
Felodipine	C791	4+3+1+1	4+3+1+1	4+4+0+0	12+10+2+2	6.5
	C792	3+3+2+0	3+3+1+1	3+3+2+1	9+9+5+2	6.25
	C793	3+3+2+1	3+2+2+1	3+2+2+0	9+7+6+2	6
	C794	0+0+0+0	1+1+1+1	0+0+0+0	1+1+1+1	1
	C795	3+1+1+1	3+1+1+1	4+0+0+1	10+2+2+3	4.25
	C796	4+1+1+1	4+2+2+1	4+2+2+1	12+5+5+3	6.25
Imatinib	C797	3+3+0+0	2+2+1+1	3+3+1+0	8+8+2+1	4.75
	C798	3+3+0+0	3+3+1+1	4+3+2+1	10+9+3+2	6
	C799	3+2+1+1	3+2+2+1	3+3+2+1	9+7+5+3	6
	C800	1+1+1+0	1+1+1+1	1+1+1+1	3+3+3+2	2.75
	C801	4+1+1+1	4+1+1+1	4+2+2+0	12+4+4+2	5.5
	C802	1+0+0+0	1+1+1+1	2+1+0+0	4+3+1+1	1.75
Control	C803	4+4+4+4	4+4+4+4	4+4+4+4	12+12+12+12	12
	C804	3+3+2+2	3+3+3+1	3+3+3+2	9+9+8+5	7.75
	C805	4+4+3+2	4+4+3+1	4+4+3+2	12+12+9+5	9.5
	C806	4+4+4+4	4+4+4+4	4+4+4+4	12+12+12+12	12
	C807	3+3+3+1	3+3+3+2	3+3+3+2	9+9+9+5	8
	C808	4+4+4+3	4+4+4+3	4+4+4+3	12+12+12+9	11.25
Mock	X1	1+1+0+0	1+1+1+0	0+0+0+0	2+2+1+0	1.25
	X2	1+1+1+1	2+2+2+2	0+0+0+0	3+3+3+3	3
	X3	1+1+1+1	2+1+1+1	0+0+0+0	3+2+2+2	2.25
	X4	1+1+1+1	2+1+1+1	0+0+0+0	3+2+2+2	2.25
	X5	1+1+1+1	2+2+2+2	0+0+0+0	3+3+3+3	3
	X6	1+1+1+0	2+1+1+1	0+0+0+0	3+2+2+1	2
