# Supplementary material for: A SARS-CoV-2-specific CAR-T-cell model identifies felodipine, fasudil, imatinib, and caspofungin as potential treatments for lethal COVID-19
Source: Cell Mol Immunol. 2023 Mar 2;20(4):351–64. doi: 10.1038/s41423-023-00985-3 (PMC9979130; doi:10.1038/s41423-023-00985-3)
Supplement: Supplementary file 3 — Supplementary figure legends [file 41423_2023_985_MOESM3_ESM.docx]

**Supplementary figure legends**

**Figure S1. SARS-CoV-2-S CAR-T cells cannot be activated by Omicron-293T cells.**

**(A)** The anti-RBD antibody was purified and diluted to different concentrations and then incubated with VSV-dG-SARS-CoV-2-Sdel18 pseudoviruses encoding wild-type spike (S) protein and its variants, including Beta, Delta, and Omicron (multiplicity of infection, MOI = 0.05), for an hour before being added to BHK21-hACE2 cells. The IC_50_ was analyzed by nonlinear regression (four-parameter) based on the eGFP fluorescence fused in viruses.

**(B)** SARS-CoV-2-S CAR-T cells were incubated with control 293T or 293T cells transfected with the mutated S protein from the Omicron variant (Omicron-293T cells) at a ratio of 3:1 for two days, and T cells in suspension were separated from adherent 293T cells and costained with anti-CD3-APC together with anti-CD69-PE or anti-CD25-FITC followed by flow cytometry analysis.

**(C)** SARS-CoV-2-S CAR-T cells were incubated with control 293T or Omicron-293T cells and then maintained in culture medium for the indicated durations. The number of viable cells was counted (mean ± s.e.m).

**Figure S2. The expression of representative cytokine genes was examined by RT‒qPCR analysis in CAR-T and S-293T cells.**

(**A**, **B**) SARS-CoV-2-S CAR-T cells were incubated with S-293T cells at a ratio of 3:1 for 48 hours, and then T cells in suspension (**A**) and adherent S-293T cells (**B**) were collected separately, followed by RT‒qPCR analysis to examine the expression of genes as indicated (Student’s t test, unpaired, two-tailed, **P* < 0.05; ***P* < 0.01; ****P* < 0.001).

**Figure S3. Inert responses of SARS-CoV-2-S CAR-T cells to antigens.**

Representative fluorescence images from cells as described in Fig. 1J at 96 h are shown.

**Figure S4. The mean (± SD) and significance test for RT‒qPCR results are shown in Fig. 2.**

The normalized values (mean ± SD) and statistical significance (Student’s t test, unpaired, two-tailed, **P* < 0.05; ***P* < 0.01; ****P* < 0.001) for data shown in Fig. 2A (A), 2B (B), and 2E (C).

**Figure S5. Cytokine release from SARS-CoV-2-S CAR-T cells incubated with Delta-293T or S-hACE2-BEAS-2B** **cells.**

**(A)** THP-1, 293T or S-293T or Delta-293T, and SARS-CoV-2-S CAR-T cells were mixed at a ratio of 10:1:10 for three days before measuring the secretion of IL8 and IFNγ (mean ± s.e.m, ****P* < 0.001).

**(B)** The expression of human ACE2 (hACE2) on BEAS-2B or BEAS-2B with hACE2 overexpression (hACE2-BEAS-2B) cells was detected using the anti-human ACE2 antibody conjugated with Alexa Fluor® 647. BEAS-2B cells without staining served as the blank.

(**C**) BEAS-2B or hACE2-BEAS-2B cells were infected with VSV-dG-SARS-CoV-2-Sdel18 viruses, and eGFP expression was measured by fluorescence imaging. Bright field is also shown.

**(D)** THP-1, hACE2-BEAS-2B or S-hACE2-BEAS-2B and SARS-CoV-2-S CAR-T cells were mixed at the indicated ratios for three days before measuring the secretion of IL8 and IFNγ (mean ± s.e.m, ****P* < 0.001).

**Figure S6. Felodipine, fasudil, imatinib, and caspofungin** **are effective in suppressing** **cytokine release from SARS-CoV-2-S CAR-T cells incubated with Delta-293T or S-hACE2-BEAS-2B** **cells.**

**(A)** THP1, Delta-293T, and SARS-CoV-2-S CAR-T cells (10:1:10) were coincubated and treated with or without felodipine, fasudil, imatinib, or caspofungin (10 μM) for three days before measuring the secretion of IL8 and IFNγ (mean ± s.e.m, ****P* < 0.001).

(**B**) THP1, S-hACE2-BEAS-2B, and SARS-CoV-2-S CAR-T cells (10:1:10) were coincubated and treated with or without felodipine, fasudil, imatinib, or caspofungin (10 μM) for three days before measuring the secretion of IL8 and IFNγ (mean ± s.e.m, ****P* < 0.001).

**Figure S7. Histological analysis of the lung lobes for hamsters treated with or without drugs**

**(A)** Representative H&E staining images for the lung lobe sections collected from hamsters as described in Fig. 5A at Day 7 post infection are shown.

**(B)** The mean (± s.e.m) (upper panel) and significance test (Student’s t test, unpaired, two-tailed) (bottom panel) for RT‒qPCR results shown in Fig. 5G.

**Figure S8. Virological analysis of the nasal turbinate, trachea, and lung tissues for hamsters treated with or without drugs**

**(A)** The levels of viral RNA, represented by SARS-CoV-2 open reading frame 1ab (ORF1ab) (left panel) and nucleocapsid protein (NP) (right panel), in the nasal turbinate, trachea, and lung tissues collected from hamsters at Day 7 post infection were measured by RT‒qPCR. Significance was calculated using one-way ANOVA (ns: nonsignificant).

**(B)** Lung tissues collected from hamsters at Day 7 post infection were subjected to RT‒qPCR analysis to examine the expression of NF-κB2. Data presented are the normalized value to the mock group after normalization to the expression of $\beta$*-actin* (Student’s t test, unpaired, two-tailed, **P* < 0.05; ***P* < 0.01; ****P* < 0.001).

**Supplementary table legends**

**Table S1.** **The comprehensive pathological score for the lung lobes in hamsters treated with or without drugs.** A comprehensive pathological score based on alveolar septum thickening and consolidation, hemorrhage, exudation, pulmonary edema and mucous, and recruitment and infiltration of inflammatory cells for lung lobes is shown.
